# Associations of fatty acids composition and estimated desaturase activities in erythrocyte phospholipids with biochemical and clinical indicators of cardiometabolic risk in non-diabetic Serbian women: the role of level of adiposity

**DOI:** 10.3389/fnut.2023.1065578

**Published:** 2023-07-20

**Authors:** Ivana Šarac, Jasmina Debeljak-Martačić, Marija Takić, Vuk Stevanović, Jelena Milešević, Milica Zeković, Tamara Popović, Jovica Jovanović, Nevena Kardum Vidović

**Affiliations:** ^1^Centre of Research Excellence in Nutrition and Metabolism, Group for Nutrition and Metabolism, Institute for Medical Research, National Institute of Republic of Serbia, University of Belgrade, Belgrade, Serbia; ^2^Department of Occupational Health, Faculty of Medicine, University of Niš, Niš, Serbia

**Keywords:** desaturase, fatty acids, cardiometabolic risk, obesity, metabolic syndrome, adiposity, lipid, women

## Abstract

**Introduction:**

Fatty acids (FAs) composition and desaturase activities can be altered in different metabolic conditions, but the adiposity-independent associations with clinical and biochemical indicators of cardiometabolic risk are still unclear. This study aimed to analyze the associations of FAs composition and estimated desaturase activities with anthropometric, clinical, and biochemical cardiometabolic risk indicators in non-diabetic Serbian women, and to investigate if these associations were independent of the level of adiposity and other confounders.

**Methods:**

In 76 non-diabetic, otherwise healthy Serbian women, aged 24-68 years, with or without metabolic syndrome or obesity (BMI=23.6±5.6 kg/m2), FA composition in erythrocyte phospholipids was measured by gas-liquid chromatography. Desaturase activities were estimated from product/precursor FAs ratios (D9D:16:1n-7/16:0; D6D:20:3n-6/18:2n-6; D5D:20:4n-6/20:3n-6). Correlations were made with anthropometric, biochemical (serum glucose, triacylglycerols, LDL-C, HDL-C, ALT, AST, and their ratios) and clinical (blood pressure) indicators of cardiometabolic risk. Linear regression models were performed to test the independence of these associations.

**Results:**

Estimated desaturase activities and certain FAs were associated with anthropometric, clinical and biochemical indicators of cardiometabolic risk: D9D, D6D, 16:1n-7 and 20:3n-6 were directly associated, while D5D and 18:0 were inversely associated. However, the associations with clinical and biochemical indicators were not independent of the associations with the level of adiposity, since they were lost after controlling for anthropometric indices. After controlling for multiple confounders (age, postmenopausal status, education, smoking, physical activity, dietary macronutrient intakes, use of supplements, alcohol consumption), the level of adiposity was the most significant predictor of desaturase activities and aforementioned FAs levels, and mediated their association with biochemical/clinical indicators. *Vice versa*, desaturase activities predicted the level of adiposity, but not other components of cardiometabolic risk (if the level of adiposity was accounted). While the associations of anthropometric indices with 16:1n-7, 20:3n-6, 18:0 and D9D and D6D activities were linear, the associations with D5D activity were the inverse U-shaped. The only adiposity-independent association of FAs profiles with the indicators of cardiometabolic risk was a positive association of 20:5n-3 with ALT/AST ratio, which requires further exploration.

**Discussion:**

Additional studies are needed to explore the mechanisms of the observed associations.

## 1. Introduction

Since 1975 the prevalence of obesity has nearly tripled worldwide (1), and it is estimated that up to 2030, 38% of the world adult population will be overweight, while 20% will be obese (2). The main culprit for the present obesity pandemic is the modern lifestyle, which includes the lack of physical activity and increased availability and consumption of energy-dense (high-fat/sugar), processed, “western-style” food (2). The increasing prevalence of overweight and obesity is an emerging public health concern not only in the western, industrialized, high-income countries, but also (and even more) in the low-income and middle-income countries (3). Particularly the countries in economic transition are on the increased burden, since their economic transition is also followed by corresponding lifestyle and dietary changes (so called “nutritional transition”) (4). Serbia is one of the European countries in transition, and during the last 50 years an increased trend in prevalence of overweight and obesity among adults, adolescents and children has been observed (5). According to the latest national survey in 2019, even 57.1% of citizens 15 years and more old in Serbia were with overweight or obesity (6), which is much higher than the average prevalence of overweight and obesity worldwide (1).

Even though the modern lifestyle is the main culprit for development of obesity and related co-morbidities, there are additional factors which can contribute to their development, including hereditary factors (7).

Overweight and obesity are among the main factors responsible for the development of the metabolic syndrome (MetS), which is the key element in development of their metabolic and cardiovascular co-morbidities. Metabolic syndrome is characterized by abdominal and visceral adiposity, insulin resistance, hyperinsulinemia, hyperglycemia, dyslipidemia [increased triacylglycerols (TAG) and decreased high-density lipoproteins - cholesterol (HDL-C) levels], hypertension, non-alcoholic fatty liver disease (NAFLD) and proinflammatory and prothrombotic state (8, 9). However, not all obesity phenotypes are connected with development of MetS, and there is also a metabolically healthy phenotype of obesity (MHO), which does not have the adverse cardiometabolic components of MetS, despite increased adiposity (10). Vice versa, MetS, associated with substantial insulin resistance and increased cardiometabolic risk, is also often observed in normal-weight or underweight subjects, despite normal (11) or even decreased adiposity (e.g., in lipodystrophic syndromes, where in particular peripheral adipose tissue is absent, while central and visceral adiposity can exist) (12, 13). Therefore, increased adiposity and components of metabolic syndrome do not have to be necessary connected (14, 15). Nevertheless, increased adiposity itself certainly worsens all the MetS components (16). In accordance, there is considerable evidence that even in the subjects with MHO, with time the MetS components and increased risk for cardiovascular disease and diabetes develop, and this is mostly determined by the increased visceral adiposity (17, 18). Vice versa, even moderate weight loss (5–10%) significantly reduces almost all of the MetS components in subjects with overweight or obesity, deepening on the initial body weight (BW), with the greater weight loss being associated with the greater MetS components reduction (19).

In the last several decades, there is substantial evidence that long chain fatty acids (FAs) compositions in various body compartments (e.g., the liver, muscle, adipose tissue, whole blood, plasma, erythrocytes, thrombocytes, cerebrospinal fluid, follicular fluid, seminal plasma, etc.) and activities of enzymes involved in their metabolism – desaturases and elongases, are connected with development of obesity (20–24), abdominal adiposity (25–27), insulin resistance and hyperglycemia (28–30), dyslipidemia (31), MetS (32–39), NAFLD (40), diabetes (41–46), and various cardiovascular diseases (hypertension, atherosclerosis, stroke, myocardial infarction, heart failure, etc.) (47–49).

The activities of enzymes involved in metabolism of FAs and profiles of FAs in erythrocytes, thrombocytes and various plasma lipids – phospholipids (PL), cholesterol-esters (CE), TAG and free fatty acids (FFA) are influenced by many factors, including genetics (50–52), gender (53–55), ethnicity (55–57), age (53, 58, 59), hormonal status (60–63), long-term and short-term dietary intakes of specific macronutrients (51, 64–67) and micronutrients (minerals: Fe, Zn; vitamins: D, B9, B12, B6, A, C; polyphenols, etc.) (64, 68–74), physical activity (53, 75), smoking (53, 74), but also (as mentioned above) by the presence of some metabolic conditions (including obesity, dyslipidemia, metabolic syndrome, NAFLD and diabetes), or by the use of certain drugs (e.g., statins) (76).

Delta-9 desaturase (D9D, also known as stearoyl-CoA desaturase 1, SCD-1), delta-6 desaturase (D6D) and delta-5 desaturase (D5D) are enzymes involved in desaturation process of, correspondingly, saturated (D9D) or polyunsaturated (D6D and D5D) long chain FAs. They are encoded by genes *Scd1*, *Fads2* and *Fads1*, respectively. To directly measure the activities of desaturases in different body compartments (specific tissues, whole blood, plasma, erythrocytes, etc.) is difficult and demanding, requires biopsy of tissues (e.g., the liver, fat tissue, muscle) and complicated techniques for the microsomal activity and expression measurements, or includes very costly and laborious methods with stable isotope administration (D_2_O, ^13^C-acetate or ^13^C-labeled FAs), which are impractical for large samples (77–79). For all of these reasons, desaturase activities are the most often estimated through their product/precursor FAs ratios in the circulation.

The activity of D9D can be estimated through the ratios 16:1n-7/16:0 and 18:1n-9/18:0 (for D9D-16 and D9D-18 activities, respectively). Similarly, the ratios 18:3n-6/18:2n-6 and 18:4n-3/18:3n-3 can both be used as the estimations of D6D activity, while the ratios of 20:4n-6/20:3 n-6 and 20:5n-3/20:4n-3 as the estimations of D5D activity, as n-3 and n-6 FAs compete for the same enzymes. However, the levels of 16:1n-7, 18:3n-6 and 20:3n-6 are more often used for the estimation of enzymatic activity, since they are less dependent on FAs dietary intakes, given that they are scarce in habitual diets, and therefore more reflect the endogenous synthesis and metabolism (20, 77, 80). Additionally, because of very rapid conversion of 18:3n-6 by elongase 5 (Elov5) to 20:3n-6 (80), the ratio of 20:3n-6 to 18:2n-6 is more often used to estimate D6D activity (49).

Erythrocyte membrane PL FAs composition is determined by a combination of diet and endogenous metabolism (61). Even though each lipid pool has a distinctive pattern of FAs profiles, there is a significant correlation between erythrocyte PL and TAG FAs composition, and hepatic, adipose tissue, muscle and plasma lipids FAs composition, because there is an exchange of FAs between different lipid pools (21, 77, 81–89). It is commonly thought that the profiles of FAs in erythrocyte membranes are more steady long-term and less influenced by the temporary dietary changes in intakes of FAs and carbohydrates, compared with the FAs profiles in plasma lipids (90, 91), even though there are some studies which disagree with this (85, 87, 89). Moreover, they are less influenced by the plasma TAG levels, compared with the whole plasma FAs profiles (92). Therefore, they can be viewed as indicators of average macronutrient intakes during several proceeding weeks or months, as well as indicators of endogenous metabolism in other tissues, particularly in the liver (90, 93, 94). Besides, there are also membrane-bond desaturases in erythrocytes, and their activity can be stimulated by oxidative stress and inflammation, caused by exercise, smoking, aging or other pro-oxidative and pro-inflammatory conditions (including obesity), in order to improve erythrocyte-membrane flexibility and protect erythrocytes from the free radical–induced hemolysis (95, 96).

Even though there is enough evidence that FAs composition (as well as the activities of desaturases and elongases) in different lipid compartments are associated with development of obesity, MetS, dyslipidemia, NAFLD, diabetes, atherosclerosis and cardiovascular diseases, the majority of the studies usually did not distinguish the associations with biochemical and clinical cardiometabolic risk markers from the associations with the increased adiposity (i.e., overweight and obesity). It is well known that the level of adiposity is in a direct association with biochemical and clinical markers of cardiometabolic risk, including levels of glucose (GLU), lipids, liver enzymes and blood pressure (BP), so it is unclear if there are some additional, adiposity-independent associations of FAs profiles and desaturase activities with biochemical and clinical indicators of cardiometabolic risk. Just a few studies controlled these associations, and only for body mass index (BMI) as a measure of adiposity (32, 97–99), while the other indicators of adiposity (100) were not considered. Some of these studies included only subjects with obesity (98), while the associations in normal-weight and underweight subjects were rarely examined. Additionally, in some studies the influence of other possible confounding factors, including sex, ethnicity, age, diet, smoking, physical activity, and alcohol consumption was not controlled. Many studies included either a mixed sample of men and women (without a clear separation of the results by sex) (39, 101) or only men (even elderly men) (32, 99), while it is known that FAs profiles and activities of desaturases can be influenced by sex (53, 54, 98), and the studies in women are particularly lacking. There are also huge racial and ethnic differences in FAs profiles and estimated activities of desaturases, which are not necessarily related to different cultural and dietary influences (55–57, 102–105). Moreover, many studies were performed in subjects with present cardiometabolic conditions (diabetes, cardiovascular diseases), while subtle changes in healthy subjects were not often examined.

### 1.1. The aim of study

This study aims to analyze the associations of FAs profiles in erythrocyte membranes PL and the estimated desaturase activities with anthropometric, clinical, and biochemical indicators of cardiometabolic risk in a cohort of non-diabetic, otherwise healthy Serbian women, and to investigate if the associations with clinical and biochemical indicators are independent of the level of adiposity, macronutrient intake, and other possible confounders.

## 2. Materials and methods

### 2.1. Participants and study design

This cross-sectional study included 76 non-diabetic and otherwise healthy Serbian women (aged 22–68 years), with or without metabolic syndrome or obesity (BMI 16.2–43.2 kg/m^2^, average 23.6 ± 5.6 kg/m^2^). The study was conducted according to the principles of the Declaration of Helsinki, and was approved by the Institute of Occupational Health Niš Ethics Board. Participants were recruited through the advertisements posted in medical services, social networks and through personal contacts of researchers. All subjects signed written informed consent before participating in the study, and completed a general questionnaire, with data included on age, postmenopausal status, education level, presence of acute and chronic illnesses, the current use of medications and supplements, smoking habit, alcohol consumption and physical activity. The subjects who did not fulfill the inclusion criteria, or met the exclusion criteria, were not enrolled in the study. The inclusion criteria were: female sex, age 20–70 years, while the exclusion criteria were presence or history of diabetes and major cardiovascular diseases (coronary heart disease, stoke), presence of other chronic or acute diseases, pregnancy and breastfeeding, recent BW changes or dietary changes, current use of hypotensive, hypolipemic or hypoglycemic drugs or drugs to treat obesity, current use of hormonal replacement therapy (HRT), oral contraceptives or corticosteroid drugs, use of 18:3n-6 supplements, and heavy alcohol consumption. Obesity, MetS, dyslipidemia or hypertension, if they were not connected with diabetes or major cardiovascular events or pharmacological treatment with of hypotensive, hypolipemic or hypoglycemic drugs, were not considered as exclusion criteria. Postmenopausal status (defined as the absence of a menstrual period for 1 year or more, or surgical menopause) was not an exclusion criterion, since the post-menopausal status was not connected with significant changes in FA profiles and desaturase activities in one study (106). Nevertheless, in our analyses we controlled for the postmenopausal status and all the analyses were also repeated with exclusion of postmenopausal women (*N* = 14). Similarly, the irregular uses of n-3 FAs supplements or Zn supplements (107), or both were not exclusion criteria, since: (1) in the analyses of D6D and D5D activities we did not used the ratios of n-3 FAs; (2) all the analyses were later controlled for n-3 FAs and Zn supplements intake; and (3) all the analyses were also repeated with exclusion of these subjects (*N* = 13).

### 2.2. The required sample size calculations

The required study sample for correlation analyses was calculated according to our pilot study in 40 non-diabetic women (aged 24–68 years, BMI = 25.5 ± 6.0 kg/m^2^). This pilot study revealed correlation coefficients *r* of erythrocytes PL FAs composition and estimated desaturase activities with examined anthropometric and biochemical indicators of cardiometabolic risk in the range of 0.324 ≤ *r* ≤ 0.693. Therefore, the required minimum sample size was 65, calculated according to formula *N* = [(*Z_α_* + *Z_β_*)/*C*]^2^ + 3, where *α* = 0.05, *β* = 0.20, *Z_α_* = 1.9600, *Z_β_* = 0.8416, *C* = 0.5*ln[(1 + *r*)/(1 − r)], and *r* = 0.324 (the minimal significant correlation coefficient observed in our pilot study) (108). To further increase the power of study and to allow for possible drop-out during analytical procedures, we increased the number of recruited participants to 80. Nevertheless, 4 subjects were later excluded from the statistical analyses due to low quality of samples for gas–liquid chromatography.

Before analyses, subjects fasted for at least 12 h and refrained from strenuous exercise and alcohol consumption for 24 h. They attended the research facility in 8:00 am for anthropometric and BP measurements, and blood collection.

### 2.3. Anthropometric and BP measurements

Measurements of body height (BH), BW, fat mass (FM), fat mass percentage (FM%), fat-free mass (FFM), waist circumference (WC), and hip circumference (HC) were performed according to standardized procedures and techniques (109, 110). Body weight, FM, FM%, and FFM were measured on a portable semiprofessional 8-electrode bioimpedance analyzer Tanita Inner Scan V BC-545 N Segmental Body Composition Monitor (Tanita, Aerolit d.o.o., Belgrade, Serbia), according to the recommendations provided by the manufacturer. Visceral fat level (VFL) was calculated according to the Tanita algorithm (111). Body mass index (kg/m^2^) was calculated, and according to their BMI, the participants were classified into four categories: underweight (BMI <18.5 kg/m^2^), normal weight (BMI: 18.5–24.9 kg/m^2^), overweight (BMI: 25–29.9 kg/m^2^), and obese (BMI ≥30 kg/m^2^) (112). Abdominal obesity was defined by WC ≥88 cm (110). Waist to hip ratio (WHR) and waist to height ratio (WHtR) were also calculated, with WHR ≥0.86 and WHtR ≥0.50 being indicative of truncal fat distribution, i.e., centripetal obesity (110, 113).

Blood pressure measurements were performed according to standardized procedures, after resting for 10 min, with a calibrated mechanic sphygmomanometer and stethoscope (Becton Dickinson, Franklin Lakes, NJ, United States), and the average of two measurements was presented as the result. Hypertension was defined by systolic blood pressure (SBP) ≥140 mmHg and/or diastolic blood pressure (DBP) ≥90 mmHg, while pre-hypertension was defined by SBP between 130 and 140 mmHg and/or DBP between 85 and 90 mmHg (114).

### 2.4. Laboratory biochemical analyses of blood samples and criteria for definition of MetS

After the anthropometric and BP measurements were performed, fasting blood samples were collected in specific Vacutest Kima® blood collection tubes, available from Vacutest Kima® S.R.L., Italy (Yunycom d.o.o., Belgrade, Serbia): Vacutest® Serum Separator Clot Activator 6 mL tubes, to assess the levels of serum GLU, TAG, low-density lipoproteins (LDL)-C, HDL-C, aspartate aminotransferase (AST), and alanine aminotransferase (ALT); and Vacutest® K3EDTA 6 mL tubes, to assess the FAs profiles in erythrocytes.

After allowing the serum coagulation tubes to clot for 30 min at room temperature, they were centrifuged at 3,000× *g* for 10 min. Following centrifugation, standard photometric assays were performed using an ARCHITECT c8000 Abbott clinical chemistry analyzer (Abbott Laboratories S.A., Belgrade, Serbia), and commercially available kits (Abbott Laboratories S.A., Belgrade, Serbia). Intra- and inter-assay coefficients of variation (CVs) for all measurements were < 5%.

The criteria for the existence of diabetes and impaired fasting glucose (IFG) were set according to the American Diabetes Association guidelines (i.e., fasting GLU ≥7.0 mmol/L and ≥ 5.6 mmol/L, respectively) (115). The existence of dyslipidemia was established based on the National Cholesterol Education Program—Adult Treatment Panel III criteria, which defined cut-off values for TAG (≥1.69 mmol/L), LDL-C (≥3.34 mmol/L), and HDL-C (<1.3 mmol/L) (116). The atherogenic risk was also calculated as the ratio of TAG/HDL-C, with cut-off ratio ≥ 1.18 (117). Metabolic syndrome was defined according to the American Heart Association/National Heart, Lung, and Blood Institute criteria (9).

### 2.5. Analysis of FAs profiles in erythrocytes PL by gas–liquid chromatography

After collecting blood into EDTA-containing vacutainer tubes, tubes were placed in the fridge at +4°C up to 1 h, until plasma and erythrocytes were separated by centrifugation at 3,000 × g for 10 min at +4°C, and erythrocytes were washed by the standard procedure (118). The aliquots of plasma and washed erythrocytes were kept frozen at −80°C until further analyses. Plasma and erythrocyte samples for FAs analysis are stable for up to 4 years when stored at −80°C (118), but analyses were performed within 6 months.

The total lipid extracts from erythrocytes were isolated by mixture of chloroform/isopropanol (7:11, v/v) by the method of Rose and Oklander (119), with addition of 2,6-di-tert-butyl-4-methylphenol (BHT, 10 mg/100 mL) as an antioxidant. The separation of PL from other lipid subclasses was done on a silica thin-layer chromatography plate using the solvent system of petroleum ether, diethyl ether and glacial acetic acid (87:12:1, v/v/v). The development time for plates was 45 min, after which they were air dried for 15 min at room temperature in a fume hood situated in a dark place. The appropriate areas of PL fraction in silica gel were rapidly scraped into screw-capped glass tubes for transmethylation. Fatty acid methyl esters (FAME) were prepared as described previously (72, 120): 1.5 mL of hexane (with added BHT, 5 mg/100 mL) and 0.2 mL of 2 M NaOH in methanol were added and tubes were heated at 85°C for 1 h, than 0.2 mL of 1 M H_2_SO_4_ in methanol was added and tubes were heated at 85°C for 2 h. After cooling to room temperature and centrifugation on 1860× *g*, for 15 min, the hexane layer was dried under a stream of N_2_. Prepared FAME were dissolved in 20 μL of heptane, and 1 μL of sample was injected into the gas–liquid chromatograph (Shimadzu GC-2014, Shimadzu Co. Ltd, Kyoto, Japan), equipped with a flame ionization detector and Rtx 2,330 column (60 m × 0.25 mm ID, film thickness of 0.2 μm, RESTEK, Bellefonte, PA, USA), with a split ratio 20:1. The chromatographic conditions were: the flow of air was 320 mL/min, the flow of H_2_ was 30 mL/min and the flow of He (carrier gas) was 1 mL/min. The temperature of the flame ionization detector was 260°C and the temperature of the injection port was 220°C. The column initial temperature was 140°C, held for 5 min, then increased at a rate of 3°C/min to 220°C, which was kept for 20 min. The quality control samples were run before the study samples and after every 20 study samples. The identification of FAME (from C:16 to C:22) was made by comparing the sample peak retention times with the certified calibration standards mixtures (PUFA-2, Supelco, Bellefonte, PA, USA, and Supelco 37 FAMEs mix + C22:5n3 FAME, Sigma Chemical Co., St. Louis, MO, USA). Finally, individual FAs were expressed as a percentage of total identified FAs. The typical FAME chromatogram of our samples was presented in Supplementary Figure S1.

The activities of D9D, D6D and D5D were estimated from the product/precursor FAs ratios (D9D:16:1n-7/16:0; D6D:20:3n-6/18:2n-6; and D5D:20:4n-6/20:3n-6). As explained in the Introduction, since 18:1n-9 and 18:0 levels and their ratio can be more influenced by dietary intakes, we chose 16:1n-7/16:0 ratio for the estimation of D9D activity (21). Total saturated FAs (SFA) were defined as a sum of 16:0 and 18:0. Total monounsaturated FAs (MUFA) were defined as sum of 16:1n-7, 18:1n-9 and 18:1n-7. Total n-6 polyunsaturated FAs (PUFA) were defined as a sum of 18:2n-6, 20:3n-6, 20:4n-6 and 22:4n-6, while total n-3 PUFA were defined as a sum of 18:3n-3, 20:5n-3, 22:5n-3, and 22:6n-6. Omega 3 index (n-3 index) was calculated as a sum of 20:5n-3 and 22:6n-3 (121). The unsatisfactory n-3 index was defined as ≤4 (very low) and 4–6 (low), while satisfactory as 6–8 (moderate) and > 8 (optimal) (83). The unsatisfactory n-6/n-3 PUFA ratio was defined as >4 (122).

### 2.6. Assessment of dietary intakes

In accordance with the European Food Safety Authority (EFSA) EU Menu methodology (109), dietary intake data were obtained by a trained medical doctor using a food propensity questionnaire (FPQ) and the twice repeated 24 h-recall method, applying the validated national Food Atlas for Portion Size Estimation (123). The average intakes of energy and macronutrients (protein, carbohydrates, total fat, cholesterol, SFA, MUFA, PUFA and *trans* FA- TFA) were calculated from the 24 h-recalls, by using the DIET ASSESS & PLAN (DAP) software (124) and the Serbian Food Composition Database, which was developed in compliance with EuroFIR standards (125). Since we did not have a validated food frequency questionnaire (FFQ) for total fat and specific dietary fats intakes, we used the EPQ to estimate frequency of consumption of specific sources of dietary fats (particularly intakes of dairy products, meat and poultry, fish, specific edible fats and oils, nuts and seeds).

### 2.7. Statistical analysis

Statistical analyses were performed using the SPSS 22.0 (SPSS Inc., Chicago, IL, United States) statistical software. The normality of data distribution was tested by the Kolmogorov–Smirnov test. Data were presented as number and % (for nominal data), the mean ± SD (for normally distributed continuous data), and median and 25th and 75th percentiles (for non-normally distributed continuous data). Since majority of data were not normally distributed, logarithmic transformations (log 10) before statistical tests were performed, so the log-transformed data followed a normal distribution and allowed for the application of parametric statistical tests (126). Correlation between log-transformed data of FAs levels and desaturase activities with anthropometric and biochemical indicators of cardiometabolic risk, as well as daily energy and macronutrient intakes and possible confounders was assessed by the Pearson’s *r* correlation coefficients (to assess the associations between two continuous variables) and Point-Biserial *r_pb_* correlation coefficients (to assess the associations between one continuous and one dichotomous categorical variable). Partial correlations were used when one or more controlling variables were included in the analyses of correlation of FAs levels and desaturase activities with anthropometric, clinical, and biochemical indicators. The following controlling variables were proposed as covariates/confounders: age (in years), physical activity (inactive, low-moderately active, moderately active, high-moderately active, highly active), educational level (under high-school, high school, upper-high school, university, university post-graduate), current smoking (no or yes), moderate alcohol consumption (no or yes), and n-3 FAs and/or Zn supplements consumption (no or yes). Linear stepwise regression models were made to explore the strength and independence of associations between FAs levels and desaturase activities with anthropometric, clinical, and biochemical indicators of cardiometabolic risk, independent of nutritional intakes and other above-mentioned confounders. One-way ANOVA was used to compare desaturase activities and FAs levels among different nutritional status categories (classified by BMI). The homogeneity of variances was tested by the Levene test, and the Tukey’s honestly significant difference (HSD) or the Games Howell (G-H) *post hoc* tests were accordingly used. Stratified regression analyses were performed across separate BMI- quartiles. Locally weighted regression with the Epanechnikov kernel was applied to estimate the smoothed, non-parametric curve fitting (127). For all analyses, statistical significance was assumed at a two-tailed *p* < 0.05, but also the Bonferroni correction for multiple testing was applied (128).

## 3. Results

### 3.1. General, anthropometric, and biochemical data: descriptives and inter-correlations

The anthropometric, clinical, and biochemical characteristics of the studied population, as well correlations between anthropometric, clinical, biochemical, and general parameters are presented in Table 1 and Supplementary Table S1, respectively.

**Table 1:** Demographic, anthropometric, biochemical, and clinical (arterial BP) data of the studied women (*N* = 76).

		Descriptives	
		Mean/Median/*N*	(SD/ICR/%)
Age	(Years)	29.0	(25.0–50.0) ^¥^
Postmenopausal	Yes	14	(18.4%) ^§^
	No	62	(81.6%) ^§^
Smoking	Yes	21	(27.6%) ^§^
	No	55	(72.4%) ^§^
Physical activity level	Inactive	10	(13.2%) ^§^
	Low-moderately active	5	(6.6%) ^§^
	Moderately active	56	(73.7%) ^§^
	High-moderately active	2	(2.6%) ^§^
	Highly active	3	(3.9%) ^§^
Educational level	Post-graduate university degree	6	(7.9%) ^§^
	Graduate university degree	30	(39.5%) ^§^
	Undergraduate university degree	12	(15.8%) ^§^
	Upper-high school degree	8	(10.5%) ^§^
	High school degree	17	(22.4%) ^§^
	Did not complete high school	3	(3.9%) ^§^
Moderate alcohol consumption	Yes	44	(57.9%) ^§^
	No	32	(42.1%) ^§^
Supplementation	n-3 PUFA supple	6	(7.9%) ^§^
	Zn supple	4	(5.3%) ^§^
	n-3 PUFA + Zn supple	3	(3.9%) ^§^
Body weight	(kg)	62.2	(55.1–69.6) ^¥^
BMI	(kg/m^2^)	21.7	(20.1–25.8) ^¥^
BMI categories	Underweight	7	(9.2%) ^§^
	Normal-weight	50	(65.8%) ^§^
	Overweight	9	(11.8%) ^§^
	Obese	10	(13.2%) ^§^
FM%	(%)	29.6	(22.2–37.5) ^¥^
FM	(kg)	18.8	(12.0–26.3) ^¥^
FFM	(kg)	43.3	(41.2–46.4) ^¥^
VFL	–	3.0	(1.0–6.5) ^¥^
Waist circumference	(cm)	77.5	(73.5–87.8) ^¥^
	≥0.88 cm	18	(23.7%) ^§^
Hip circumference	(cm)	99.8	(94.0–107.5) ^¥^
WHR	–	0.8	(0.1) ^#^
	≥0.86	15	(19.7%) ^§^
WHtR	–	0.5	(0.4–0.5) ^¥^
	≥0.50	26	(34.2%) ^§^
Glucose	(mmol/L)	4.7	(4.4–4.9) ^¥^
Triacylglycerols	(mmol/L)	0.8	(0.6–1.2) ^¥^
HDL-cholesterol	(mmol/L)	1.6	(0.3) ^#^
LDL-cholesterol	(mmol/L)	2.6	(2.0–3.4) ^¥^
TAG/HDL-C	–	0.5	(0.3–0.8) ^¥^
ALT	(U/L)	15.1	(12.4–19.3) ^¥^
AST	(U/L)	18.6	(15.9–22.3) ^¥^
ALT/AST	–	0.8	(0.7–1.0) ^¥^
Systolic BP	(mmHg)	116.6	(15.0) ^#^
Diastolic BP	(mmHg)	69.4	(10.4) ^#^

^#^Mean (SD); ^¥^Median (ICR); ^§^*N* (%).

BMI, Body Mass Index; FM, Fat mass; FM%, Percentage of fat mass; FFM, Fat-free mass; VFL, visceral fat level; WHR, waist to hip ratio; WHtR, waist to height ratio; HDL, High Density Lipoprotein; LDL, Low Density Lipoprotein; TAG/HDL-C, Triacylglycerols/ HDL-C ratio; AST, aspartate aminotransferase; ALT, alanine aminotransferase; ALT/AST, alanine aminotransferase /aspartate aminotransferase ratio; BP, Blood Pressure; SD, standard deviation; ICR, interquartile range.

The mean age in the cohort was 36.6 ± 13.5 years, but above half of the subjects (51.3%) were up to 30 years old. Postmenopausal women made less than 1/5 of the study sample, and none of them used HRT. The majority of the subjects had a graduate university or high school degree, and were classified as moderately active. Less than 1/3 of the subjects were current smokers, while moderate alcohol consumption was reported by 3/5 of the subjects, with all consuming maximum 1–2 times per week up to two units of alcoholic drinks (indicating low-moderate alcohol consumption in general). Omega-3, Zn or both supplements’ use was reported only by 13 subjects, and all of them only irregularly used these supplements (not every day), and in quite low doses (20:5n-3 + 22:6n-3 no more than 300 mg/day, and Zn no more than 15 mg/day) (Table 1).

Mean BMI was 23.6 ± 5.6 kg/m^2^, varying from 16.2 to 43.2 kg/m^2^. According to BMI, about 2/3 of the subjects were normal-weight, while 1/4 were overweight/obese, and less 1/10 than were underweight. According to WC and WHR, about 1/5 had centripetal obesity, but according to WHtR, about 1/3 were centripetally obese. Centripetal obesity was present in 1/5–1/3 of the subjects (depending on whether WC, WHR or WHtR were used as indicators). A relatively small percentage of the subjects had pre-hypertension/hypertension, IFG, decreased HDL-C and increased TAG levels, while MetS was observed in 1/5 of the subjects. The most prevalent among dyslipidemias was increased LDL levels (Table 1).

As expected, most biochemical and clinical cardiometabolic risk indicators were significantly positively correlated with anthropometric indices of general and centripetal adiposity, age, menopausal status and smoking, while were negatively correlated with education and physical activity (Supplementary Table S1). Since all anthropometric, biochemical, clinical, and general data were inter-correlated (Supplementary Table S1), they were included as possible confounders in further analyses.

### 3.2. Fatty acid profiles and activities of desaturases: descriptives and inter-correlations

In Table 2 are given descriptive data on FAs profiles (expressed as a percentage of total identified FAs in erythrocytes PL) and corresponding activities of desaturases. The presented laboratory data imply that the intake of n-3 FAs in the examined population was very low: only 4 (5.3%) of the subjects had satisfactory n-3 index (>6), while even 41 (53.9%) of the subjects had very low n-3 index (<4). Correspondingly, satisfactory n-6 /n-3 PUFA ratio was observed only in 2 (2.6%) of the subjects.

**Table 2:** Fatty acid profiles in erythrocytes PL analyzed by gas–liquid chromatography.

	FA (Symbol)	Descriptives	
		Mean/Median	(SD/ICR)
SFA	16:0	21.3	(20.2–22.3) ^¥^
	18:0	20.5	(19.9–21.3) ^¥^
	Total	42.1	(2.7) ^#^
MUFA	16:1n-7	0.2	(0.2–0.3) ^¥^
	18:1n-9	13.5	(1.3) ^#^
	18:1n-7	1.6	(0.3) ^#^
	Total	15.4	(1.4) ^#^
n-6 PUFA	18:2n-6	13.7	(1.5) ^#^
	20:3n-6	1.7	(1.5–2.0) ^¥^
	20:4n-6	17.7	(16.0–18.9) ^¥^
	22:4n-6	4.2	(3.7–4.9) ^¥^
	Total	36.9	(3.0) ^#^
n-3 PUFA	18:3n-3	0.1	(0.0) ^#^
	20:5n-3	0.2	(0.2–0.3) ^¥^
	22:5n-3	1.5	(1.2–1.7) ^¥^
	22:6n-3	3.6	(3.2–4.6) ^¥^
	Total	5.5	(4.8–6.3) ^¥^
	n-6/n-3 PUFA ratio	6.8	(5.7–7.8) ^¥^
	n-3-index (20:5n-3 + 22:6n-3)	3.9	(3.4–4.8) ^¥^
Estimated desaturases activities	D9D 16:1n-7/16:0 × 10^3^	11.5	(9.7–13.1) ^¥^
	D6D 20:3n-6/18:2n-6 × 10^2^	12.2	(10.8–14.4) ^¥^
	D5D 20:4n-6/20:3n-6	10.3	(2.3) ^#^

^#^Mean (SD); ^¥^Median (ICR).

FA, fatty acid; PL, phospholipids; SFA, saturated fatty acids; MUFA, monounsaturated fatty acids; PUFA, polyunsaturated fatty acids; 16:0, palmitic acid; 18:0, stearic acid; 16:1n-7, palmitoleic acid; 18:1n-9, oleic acid; 18:1n-7, cis-vaccenic acid; 18:2n-6, linoleic acid; 20:3n-6, di-homo-gamma-linolenic acid; 20:4n-6, arachidonic acid; 22:4n-6, adrenic acid; 18:3n-3, alpha-linolenic acid; 20:5n-3, eicosapentaenoic acid; 22:5n-3, docosapentaenoic acid; 22:6n-3, docosahexaenoic acid; D9D, delta-9 desaturase (i.e., D9D-16; stearoyl-CoA desaturase 1, SCD-1); D6D, delta-6 desaturase; D5D, delta-5 desaturase; SD, standard deviation; ICR, interquartile range. Individual FAs were expressed as a percentage of total identified FAs.

In Supplementary Table S2 are given inter-correlations of FAs profiles and activities of desaturases. Estimated activity of D5D was in significant negative inter-correlations with activities of D9D and D6D (*r* = −0.289, *p* < 0.05 and *r* = −0.626, *p* < 0.001, respectively), while activities of D9D and D6D were in a significant positive inter-correlation (*r* = 0.384, *p* < 0.001). The particularly strong negative inter-correlation between D6D and D5D activities remained significant even after multiple adjustments for BMI, age, smoking status, physical activity, educational level, moderate alcohol consumption and n-3 PUFA and Zn supplementation (*r* = −0.593, *p* < 0.001).

There was also a significant inter-correlation between different FAs (Supplementary Table S2). In general, both SFA (more) and MUFA (less) correlated negatively with proportions of n-6 (more) and n-3 PUFA (less). Pearson coefficients of SFA and MUFA correlations with n-6 PUFA were, respectively, *r* = −0.845 (*p* < 0.001) and *r* = −0.521 (*p* < 0.001), while with n-3PUFA were, respectively, *r* = −0.395 (*p* < 0.001) and *r* = −0.274 (*p* < 0.05). The adjustments for multiple confounders did not change these results. In fact, the correlation coefficients become even slightly higher: *r* = −0.857 (*p* < 0.001), *r* = −0.556 (*p* < 0.001), *r* = −0.397 (*p* < 0.001) and *r* = −0.282 (*p* < 0.05).

### 3.3. Correlations of FAs profiles and activities of desaturases with anthropometric, biochemical, clinical, and general data

In Table 3 are presented correlations of FAs profiles and desaturase activities with general, anthropometric, clinical (BP) and biochemical parameters, before adjustment for confounders. In Table 4 are presented correlations of selected FAs (18:0, 16:1n-7, 20:3n-6 and 20:5n-3) and desaturase activities with clinical and biochemical parameters, after adjustment for confounders. As confounders were used: (1) Model 1 – only BMI; (2) Model 2 – BMI and age; (3) Model 3 – all confounders: BMI, age, educational level, smoking status, physical activity, moderate alcohol consumption, and n-3 PUFA and Zn supplementation (Note: due to high inter-correlation of postmenopausal status and age, postmenopausal status was not included in the Model 3 as a confounder, to avoid multicollinearity).

**Table 3:** Correlations of FAs profiles and desaturase activities with general, anthropometric, biochemical, and clinical (BP) parameters, without adjustment for confounders.

	SFA		MUFA			n-6 PUFA				n-3 PUFA				Estimated desaturases activities		
	16:0	18:0	16:1n-7	18:1n-9	18:1n-7	18:2n-6	20:3n-6	20:4n-6	22:4n-6	18:3n-3	20:5n-3	22:5n-3	22:6n-3	D9D	D6D	D5D
	*r*															
Age	−0.077	−0.268^*^	0.358^**^	0.214	−0.332^**^	0.005	0.244^*^	0.048	−0.052	0.255	0.256^*^	0.222	0.066	0.401^**^	0.245^*^	−0.205
Smoking^§^	0.097	−0.184	0.270^*^	0.172	−0.170	0.133	0.237^*^	−0.145	−0.089	0.022	0.057	0.093	−0.115	0.256^*^	0.174	−0.306^**^
Post-menopause^§^	−0.229^*^	−0.105	0.139	0.055	−0.323^**^	0.042	0.168	0.113	0.047	0.260	0.150	0.152	0.060	0.215	0.149	−0.097
Physical activity	0.177	0.284^*^	−0.136	0.079	0.042	0.013	−0.287^*^	−0.117	−0.130	−0.159	−0.243^*^	−0.249^*^	−0.343^**^	−0.197	−0.298^**^	0.208
Education	0.059	0.076	−0.260^*^	−0.153	0.158	−0.101	−0.119	−0.028	−0.026	0.087	0.150	0.045	0.201	−0.292^*^	−0.070	0.097
Alcohol^§^	−0.092	−0.030	0.035	−0.016	0.153	−0.278^*^	0.067	0.292^*^	0.260^*^	−0.005	−0.198	0.083	−0.181	0.065	0.208	0.099
n-3 FA suppl. ^§^	0.151	−0.071	0.291^*^	0.220	−0.069	−0.080	0.119	−0.146	−0.123	−0.075	0.312^**^	0.139	0.030	0.262^*^	0.161	−0.194
n-3 FA + Zn suppl.	0.107	−0.104	0.297^**^	0.177	−0.119	−0.131	0.145	−0.087	−0.069	0.099	0.362^**^	0.196	0.095	0.282^*^	0.213	−0.186
BW	−0.129	−0.320^**^	0.390^**^	−0.020	−0.009	−0.133	0.418^**^	0.237^*^	0.255^*^	0.180	0.076	0.112	0.059	0.450^**^	0.491 ^**^	−0.266^*^
BMI	−0.024	−0.295^**^	0.473 ^**^	0.061	0.012	−0.136	0.435 ^**^	0.134	0.164	0.120	0.071	0.134	0.025	0.507 ^**^	0.510 ^**^	−0.339^**^
FM%	−0.170	−0.304^**^	0.326^**^	−0.026	0.136	−0.229^*^	0.317^**^	0.285^*^	0.224	0.173	0.137	0.155	0.200	0.395^**^	0.437 ^**^	−0.144
FM	−0.161	−0.323^**^	0.365^**^	−0.025	0.082	−0.199	0.372^**^	0.277^*^	0.246^*^	0.184	0.118	0.144	0.150	0.434 ^**^	0.478 ^**^	−0.200
FFM	−0.094	−0.220	0.246^*^	−0.078	−0.189	0.039	0.333^**^	0.172	0.220	0.037	−0.023	0.015	−0.090	0.288^*^	0.318^**^	−0.221
VFL	−0.095	−0.335^**^	0.482 ^**^	0.133	−0.086	−0.155	0.375^**^	0.173	0.125	0.293	0.176	0.179	0.091	0.538 ^**^	0.458 ^**^	−0.260^*^
WC	−0.088	−0.210	0.329^**^	−0.004	0.025	−0.194	0.383^**^	0.192	0.206	0.131	0.060	0.114	0.066	0.374^**^	0.486 ^**^	−0.257^*^
HC	−0.109	−0.279^*^	0.370^**^	0.001	0.076	−0.188	0.327^**^	0.244^*^	0.235^*^	0.149	0.058	0.101	0.075	0.424^**^	0.427^**^	−0.175
WHR	−0.021	−0.030	0.127	−0.009	−0.048	−0.110	0.270^*^	0.041	0.075	0.062	0.034	0.076	0.024	0.140	0.329^**^	−0.234^*^
WHtR	−0.009	−0.182	0.374^**^	0.053	0.038	−0.187	0.377^**^	0.110	0.132	0.086	0.054	0.126	0.038	0.398^**^	0.477 ^**^	−0.297^**^
Glucose	0.037	−0.239^*^	0.383^**^	0.175	−0.171	−0.128	0.186	0.040	0.008	−0.026	0.149	0.176	0.009	0.394^**^	0.253^*^	−0.155
Triacylglycerols	0.040	−0.286^*^	0.355^**^	0.113	0.041	−0.082	0.352^**^	0.025	0.080	−0.014	0.052	0.164	0.023	0.363^**^	0.399^**^	−0.321^**^
HDL-cholesterol	−0.064	0.045	−0.286^*^	−0.001	−0.240^*^	0.058	−0.241^*^	0.022	−0.068	−0.015	0.108	0.136	0.129	−0.282^*^	−0.274^*^	0.242^*^
LDL-cholesterol	−0.134	−0.189	0.252^*^	0.156	−0.319^**^	−0.038	0.133	0.093	−0.051	0.252	0.262^*^	0.281^*^	0.083	0.307^**^	0.154	−0.075
TAG/HDL-C	0.051	−0.235^*^	0.364^**^	0.088	0.107	−0.081	0.348^**^	0.013	0.083	−0.006	0.006	0.085	−0.023	0.369^**^	0.394^**^	−0.324^**^
ALT	0.092	0.017	0.259^*^	0.211	0.009	−0.178	−0.098	−0.068	−0.117	−0.217	0.272^*^	0.204	0.017	0.246^*^	−0.010	0.055
AST	−0.026	−0.015	0.109	−0.036	0.115	−0.233^*^	−0.025	0.113	0.063	−0.061	0.234^*^	0.118	0.131	0.123	0.092	0.087
ALT/AST	0.150	−0.242^*^	0.419^**^	0.212	−0.176	−0.089	0.115	−0.089	−0.174	0.159	0.468 ^**^	0.330^**^	0.118	0.398^**^	0.161	−0.159
Systolic BP	−0.129	−0.056	−0.031	−0.209	0.061	−0.047	0.300^*^	0.172	0.167	0.122	0.011	−0.007	0.116	0.008	0.333^**^	−0.193
Diastolic BP	0.038	−0.193	0.199	0.071	−0.035	0.089	0.167	−0.013	−0.053	−0.050	0.168	−0.030	0.092	0.201	0.129	−0.171

§ Point-Biserial r_pb_ correlation coefficients (otherwise - data represent Pearson’s correlation coefficients – *r*).

* *p* < 0.05; correlation is significant at the 0.05 level (2-tailed).

** *p* < 0.01; correlation is significant at the 0.01 level (2-tailed).

Underlined coefficients represent those which remained significant after the Bonferroni’s corrections for multiple correlations. FA, fatty acid; SFA, saturated fatty acids; MUFA, monounsaturated fatty acids; PUFA, polyunsaturated fatty acids; PA, palmitic acid; 18:0, stearic acid; 16:1n-7, palmitoleic acid; 18:1n-9, oleic acid; 18:1n-7, cis-vaccenic acid; 18:2n-6, linoleic acid; 20:3n-6, di-homo-gamma-linolenic; 20:4n-6, arachidonic acid; 22:4n-6, adrenic acid; 18:3n-3, alpha-linolenic acid; 20:5n-3, eicosapentaenoic acid; 22:5n-3, docosapentaenoic acid; 22:6n-3, docosahexaenoic acid; D9D, delta-9 desaturase (i.e., D9D-16; stearoyl-CoA desaturase 1, SCD-1); D6D, delta-6 desaturase; D5D, delta-5 desaturase; BMI, Body Mass Index; FM, Fat mass; FM%, Percentage of fat mass; FFM, Fat-free mass; VFL, visceral fat level; WC, waist circumference; HC, hip circumference; WHR, waist to hip ratio; WHtR, waist to height ratio; HDL, High Density Lipoprotein; LDL, Low Density Lipoprotein; TAG/HDL-C-R, Triacylglycerols/HDL-cholesterol ratio; AST, aspartate aminotransferase; ALT, alanine aminotransferase; ALT/AST-R, alanine aminotransferase /aspartate aminotransferase ratio; BP, Blood Pressure; r, Pearson’s correlation coefficients; p, statistical significance of correlations. Individual FAs were expressed as a percentage of total identified FAs.

**Table 4:** Correlations of selected FAs (18:0, 16:1n-7, 20:3n-6 and 20:5n-3) and desaturase activities with biochemical and clinical (BP) parameters, after adjustment for BMI and all other confounders.

	MODEL 1 (corrected for BMI)							MODEL 2 (corrected for BMI and age)							MODEL 3 (corrected for BMI, age, and all other confounders)						
	18:0	16:1n-7	20:3n-6	20:5n-3	D9D	D6D	D5D	18:0	16:1n-7	20:3n-6	20:5n-3	D9D	D6D	D5D	18:0	16:1n-7	20:3n-6	20:5n-3	D9D	D6D	D5D
	*r*							*r*							*r*						
GLU	−0.136	0.237^*^	0.011	0.132	0.237^*^	0.058	−0.019	−0.065	0.153	−0.044	0.039	0.136	0.016	0.028	−0.065	0.217	−0.030	−0.041	0.186	0.070	0.033
TAG	−0.152	0.120	0.143	0.015	0.107	0.156	−0.166	−0.073	0.008	0.097	−0.110	−0.032	0.121	−0.130	−0.077	−0.041	0.078	−0.074	−0.074	0.114	−0.101
HDL-C	−0.104	−0.092	−0.056	0.157	−0.070	−0.057	0.105	−0.117	−0.081	−0.050	0.176	−0.056	−0.052	0.100	−0.088	−0.017	−0.063	0.163	−0.007	−0.084	0.082
LDL-C	−0.090	0.094	−0.034	0.255^*^	0.148	−0.044	0.059	0.034	−0.076	−0.141	0.137	−0.042	−0.137	0.159	0.044	−0.161	−0.191	0.200	−0.106	−0.177	0.248^*^
TAG/HDL-C	−0.083	0.126	0.131	−0.042	0.108	0.141	−0.166	−0.012	0.035	0.091	−0.148	−0.003	0.110	−0.135	−0.027	−0.025	0.082	−0.116	−0.054	0.117	−0.107
ALT	0.006	0.315^**^	−0.090	0.275^*^	0.308^**^	0.011	0.045	0.138	0.217	−0.194	0.173	0.180	−0.058	0.129	0.129	0.192	−0.196	0.170	0.164	−0.068	0.150
AST	−0.007	0.109	−0.040	0.232^*^	0.127	0.091	0.102	0.016	0.082	−0.056	0.211	0.096	0.080	0.117	−0.025	0.085	−0.008	0.194	0.116	0.130	0.100
ALT/AST	−0.128	0.261^*^	−0.102	0.490 ^**^	0.219	−0.090	−0.007	−0.046	0.170	−0.180	0.438 ^**^	0.100	−0.155	0.049	−0.076	0.081	−0.189	0.473 ^**^	0.018	−0.176	0.090
SBP	0.076	−0.283^*^	0.147	−0.020	−0.258^*^	0.155	−0.062	0.000	−0.207	0.215	0.085	−0.160	0.216	−0.115	−0.019	−0.196	0.284^*^	−0.011	−0.152	0.289^*^	−0.175
DBP	−0.085	0.010	−0.010	0.153	−0.004	−0.096	−0.040	−0.079	0.001	−0.014	0.148	−0.015	−0.101	−0.036	−0.102	−0.049	0.007	0.076	−0.062	−0.084	−0.028

MODEL 1 – corrected for BMI; MODEL 2 – corrected for BMI and age; MODEL 3 – corrected for BMI, age, smoking, physical activity, level of education, moderate alcohol consumption, and n-3/Zn supplementation.

* *p* < 0.05; correlation is significant at the 0.05 level (2-tailed).

** *p* < 0.01; correlation is significant at the 0.01 level (2-tailed).

Underlined coefficients represent those which remained significant after the Bonferroni’s corrections for multiple correlations.

FA, fatty acid; 18:0, stearic acid; 16:1n-7, palmitoleic acid; 20:3n-6, di-homo-gamma-linolenic; 20:5n-3, eicosapentaenoic acid; D9D, delta-9 desaturase (i.e., D9D-16; stearoyl-CoA desaturase 1, SCD-1); D6D, delta-6 desaturase; D5D, delta-5 desaturase; BMI, Body Mass Index; GLU, glucose: TAG, triacylglycerols; HDL-C, High Density Lipoprotein-Cholesterol; LDL-C, Low Density Lipoprotein-Cholesterol; TAG/HDL-C, Triacylglycerols/ HDL-C ratio; AST, aspartate aminotransferase; ALT, alanine aminotransferase; ALT/AST, ALT/AST ratio; BP, Blood Pressure; SBP, Systolic Blood Pressure; DBP, Diastolic Blood Pressure; *r*, Pearson’s correlation coefficients; *p*, statistical significance of correlations Non-normally distributed data were log 10 transformed before analyses. Individual FAs were expressed as a percentage of total identified FAs.

According to Table 3, the levels of 16:1n-7 were in positive correlations with age, smoking, n-3, and n-3/Zn supplementation, all measures of adiposity (BMI, FM, %FM, VFL, WC, HC and WHtR), levels of GLU, TAG, LDL-C, ALT and ratios of TAG/HDL-C and ALT/AST, and in negative correlation with HDL-C and level of education. Similarly, the levels of 20:3n-6 were in positive correlations with age, smoking, all measures of adiposity and centripetal fat distribution (BW, BMI, FM, %FM, VFL, WC, HC, WHR and WHtR), SBP, levels of TAG and ratio of TAG/HDL-C, and in negative correlation with HDL-C and physical activity level. In contrast, the levels of 18:0 were in negative correlations with age, all measures of adiposity (excluding measures of centripetal fat distribution: WC, WHR and WHtR), levels of GLU, TAG and ratios of TAG/HDL-C and ALT/AST, and positive correlation with the level of physical activity. Some anthropometric indices of adiposity were also in positive correlations with levels of 20:4n-6 and 22:4n-6 (BW, FM, FM%, HC, with 20:4n-6; BW, FM, HC, with 22:4n-6), while levels of 18:3n-3 were in negative correlation with FM%. Moderate alcohol consumption was in positive correlation with 20:4n-6 and 22:4n-6, and negative correlation with 18:3n-3. Proportions of 20:5n-3 were in significant positive correlation with age, n-3 PUFA and n-3 PUFA/Zn supplementation, LDL-C, ALT, AST and ALT/AST ratio, in negative correlation with physical activity level. Also, proportions of 22:5n-3 were in positive correlation with LDL-C and ALT/AST ratio, as well as in negative correlation with physical activity level. The proportions of 22:6n-3 were in negative correlation with physical activity level (no other correlations were found). *Cis*–vaccenic acid (18:1n-7) was in negative correlation with age, postmenopausal status, LDL-C and HDL-C, while 16:0 was only in negative correlation with postmenopausal status.

The estimated activities of D9D and D6D were in positive correlations with age, almost all measures of adiposity and centripetal fat distribution (except WHR with D9D), levels of GLU, TAG, ratio TAG/HDL-C, and in negative with HDL-C. In addition, D9D activity was also in positive correlation with ALT, ALT/AST ratio, and n-3 PUFA and n-3 PUFA/Zn supplementation and negative correlation with level of education, while D6D activity was in positive correlation with SBP and negative with physical activity. In contrast, the activity of D5D was in negative correlations with measures of adiposity and centripetal fat distribution (except FM, %FM, and HC), levels of TAG and ratio TAG/HDL-C, smoking, and in positive correlation with HDL-C.

After applying the Bonferroni’s corrections for multiple correlations (which established statistical significance at *p* < 0.00012), there remained significant only correlations of 16:1n-7 with BMI and VFL, correlations of 20:3n-6 with BMI, correlations of D9D with BMI, FM, VFL, correlations of D6D with BMI, FM, FM%, VFL, WC and WHtR, as well as correlations of 20:5n-3 with ALT/AST (Table 3).

The corrections for BMI (Model 1) and BMI and age (Model 2) annulled the significance for all of the above-mentioned correlations of FAs profiles and desaturase activities with clinical and biochemical markers of cardiometabolic risk (SBP, GLU, TAG, HDL-C, LDL-C, TAG/HDL-C, ALT, ALT/AST) except for correlations of 20:5n-3 with ALT/AST ratio (Table 4). Further adjustments for other significant confounders (Model 3) did not significantly change these results, even though D5D and 20:3n-6 started to positively correlate with SBP, and D5D started to positively correlate with LDL-C. However, the Bonferroni’s corrections for multiple correlations (which established statistical significance at *p* < 0.00036) annulled significance of these correlations, and only correlations of 20:5n-3 with ALT/AST ratio remained significant (*r* = 0.473, *p* = 0.00006). The positive association of 20:5n-3 levels with ALT/AST ratio remained significant even after additional controlling for n-3 PUFA dietary intakes and fish intake and all other above-mentioned confounders, including n-3 PUFA supplementation (*r* = 0.474, *p* = 0.00013), or when subjects with n-3 PUFA or n-3 PUFA/Zn supplements use were excluded from the analysis (*r* = 0.520, *p* = 0.00007, and *r* = 0.511, *p* = 0.00015, respectively).

Very similar results were obtained when corrections were performed with FM, FM% VFL (Supplementary Table S3), or WC, HC, WHtR (Supplementary Table S4) as measures of adiposity instead of BMI, only that after multiple adjustments, apart from correlations of 20:5n-3 with ALT/AST, there also remained significant positive correlations of 20:3n-6 and D6D with SBP, and positive correlations of 16:1n-7 with GLU (but the later not in case when we controlled for VFL or HC). However, the Bonferroni’s corrections for multiple correlations (which established statistical significance at *p* < 0.00036) again annulled significance of these correlations, except for 20:5n-3 with ALT/AST ratio (Supplementary Tables S3, S4).

### 3.4. Dietary intakes

The findings of unsatisfactory n-3 index and n-6/n-3 PUFA ratio in erythrocytes PL were in accordance with the data obtained by FPQ, indicating very low consumption of fish, nuts and oils rich in n-3 FAs. Fish was consumed less than once per week by 73.7% of the subjects, while 18.4% of the subjects consumed fish even less than once per month. The majority of the studied subjects (56.6%) did not consume frequently nuts and seeds (i.e., consumed less than once per week). Only two participants consumed food (soft-margarines) enriched with n-3 FAs. The majority of the participants consumed refined sunflower oil for cooking and salads (88.2%), while olive oil was regularly used (more than 5 times per week) by 43.4% of the subjects, mostly only for salads, rarely for cooking. Other oils were very rarely used (by 13.2% of the subjects): grape seed, corn, palm, and coconut oil, while only two participants used plant oils which are rich in n-3 FAs (flaxseed and rapeseed oil). Pork lard was at least sometimes used for cooking by 53.9% of the subjects, with 22.3% of the subjects reporting very frequent use (2–3 times per week or more). Interestingly, the level of education was in negative association with use of pork lard and sunflower oil, and in positive association with use of olive oil and other oils (data not shown). Very frequent use of full-fat dairy products (more than 5 times per week) was reported by all subjects, very frequent (more than 5 times per week) consumption of red meat and poultry (and related products) was reported by 88.2% of the subjects. Therefore, the main sources of FAs in the diet were dairy products, meat and poultry, sunflower oil, and in some cases olive oil and pork lard. This indicates one diet rich in SFA, MUFA and n-6 PUFA, and very poor in n-3 PUFA (i.e., typical “western diet”).

In line with that, according to data from 24 h-recalls, fat contributed the most to total energy intake. About 2/3 of participants (64.5%) consumed more than 40% of energy as fat in their diet, and more than 1/2 of participants (51.3%) consumed less than 40% of energy in the form of carbohydrates, indicating a high-fat/low-carbohydrate dietary pattern. Particularly SFA intakes dominated, while intakes of MUFA and PUFA were lower. Omega-3 FAs intakes were negligible (Table 5).

**Table 5:** Nutritional intakes of macronutrients, according to 24 h-recalls data.

Energy / macronutrients intakes	Units	Descriptives	
		Mean/Median	SD/ICR
Total caloric intake	(kcal/d)	1827.7	(1540.7–2292.0)^¥^
Carbohydrate intake	(kcal/d)	751.2	(551.5–980.5)^¥^
Protein intake	(kcal/d)	279.5	(235.1–341.8)^¥^
Fat intake	(kcal/d)	792.6	(590.3–949.2)^¥^
SFA intake	(kcal/d)	239.2	(196.6–313.7)^¥^
MUFA intake	(kcal/d)	215.5	(172.6–310.2)^¥^
PUFA intake	(kcal/d)	211.4	(139.3–299.1)^¥^
n-6 PUFA intake	(kcal/d)	208.4	(134.5–289.4)^¥^
n-3 PUFA intake	(kcal/d)	4.0	(2.4–7.3)^¥^
TFA intake	(kcal/d)	10.8	(6.2–18.9)^¥^
Cholesterol intake	(g/d)	282.5	128.9^#^

^#^Mean (SD); ^¥^ Median (ICR).

SFA, saturated fatty acids; MUFA, monounsaturated fatty acids; PUFA, polyunsaturated fatty acids; TFA, trans fatty acids; SD, standard deviation; ICR, interquartile range.

To convert the intakes of proteins, carbohydrates, and fats into grams per day (g/d), it is needed to divide the caloric intakes of proteins and carbohydrates by 4, and the caloric intakes of fats by 4.

Interestingly, after multiple adjustments for all possible confounders (BMI, age, smoking, physical activity, alcohol consumption, educational level and n-3 PUFA and Zn supplements use), activities of desaturases did not show significant correlations with macronutrient intakes, except for protein intake with D5D activity (*r* = 0.372, *p* < 0.01). Regarding examined FAs, protein intake was negatively associated with 20:3n-6 (*r* = −0.328, *p* < 0.05), 20:5n-3 was positively associated with MUFA, n-3 PUFA, total fat and protein intake (*r* = 0.398, *r* = 0.356, *p* < 0.01, and *r* = 0.280, *r* = 0.273, *p* < 0.05, respectively), while 16:0 was negatively associated with n-6 and positively associated with n-3 PUFA intakes (*r* = −0.289 and *r* = 0.265, respectively, *p* < 0.05). Nevertheless, the Bonferroni’s corrections for multiple correlations (which established statistical significance at *p* < 0.00018) annulled significance of all these correlations. No other significant associations were found.

### 3.5. Linear regression analyses for prediction of selected FAs and estimated desaturases activities

The stepwise models for prediction of proportions of 16:1n-7, 18:0, 20:3n-6, 20:5n-3 and desaturase activities included as predictors: BMI, age, postmenopausal status, smoking, physical activity, moderate alcohol intake, educational level, n-3 PUFA and Zn supplementation, and dietary total energy intakes (in kcal/d), intakes of carbohydrates, proteins, total fats, SFA, MUFA, n-6 PUFA, n-3 PUFA, TFA and cholesterol (in g/d).

The stepwise regression models (Table 6) revealed that significant predictors for 18:0, 16:1n-7, 20:3n-6 and 20:5n-3 were (respectively): BMI (negative); BMI and age (positive); BMI (positive); dietary intakes of n-3 PUFA and n-3 PUFA and Zn supplementation. Significant predictors for D9D, D6D and D5D were (*r*espectively): BMI and age (positive); BMI (positive); BMI and smoking (negative). However, the models explained only a small percentage of the depended variables variation, less than 30% (*R^2^* adjusted), which indicates that some other factors (including genetics) significantly contribute (Table 6). In almost all of the mentioned regression analyses (except for 20:5n-3), BMI was the most significant predictor (Table 6). When only BMI was included in regression models (Supplementary Table S5), for all examined FAs and desaturases it was a significant predictor (again, except for 20:5n-3), and it explained up to 25% of their variance (the most significantly for D6D, D9D, 16:1n-7 and 20:3n-6).

**Table 6:** Linear (stepwise) regression models for prediction of proportions of 18:0, 16:1n-7, 20:3n-6, 20:5n-3 and desaturase activities in erythrocytes PL.

Dependent variable	Model	Adjusted *R^2^*	ANOVA *p*	Coefficients	*β*	*B*	95% *CI* for *B*
							Lower Bound Upper Bound
18:0	1	0.073	0.016*	(Constant)		1.459	[1.343, 1.575]**
				BMI	−0.295	−0.105	[−0.190, −0.020]*
16:1n-7	1	0.251	<0.001**	(Constant)		−1.708	[−2.157, −1.259]**
				BMI	0.401	0.573	[0.250, 0.895]**
				Age	0.235	0.201	[0.008, 0.395]*
20:3n-6	1	0.177	<0.001**	(Constant)		−0.387	[−0.707, −0.067]*
				BMI	0.435	0.453	[0.219, 0.688]**
20:5n-3	1	0.265	<0.001**	(Constant)		−0.570	[−0.636, −0.505]**
				n-3 PUFA dietary intakes	0.415	0.250	[0.115, 0.385]**
				n-3 PUFA/Zn supplementation	0.239	0.060	[0.004, 0.116]*
D9D	1	0.302	<0.001**	(Constant)		−0.065	[−0.475, 0.345]
				BMI	0.423	0.573	[0.278, 0.867]**
				Age	0.271	0.220	[0.043, 0.397]*
D6D	1	0.248	<0.001**	(Constant)		0.384	[0.083, 0.685]*
				BMI	0.510	0.523	[0.303, 0.744]**
D5D	1	0.163	0.001**	(Constant)		1.480	[1.140, 1.821]**
				BMI	−0.310	−0.339	[−0.589, −0.089]**
				Smoking	−0.273	−0.061	[−0.112, −0.010]*

MODEL 1 – predictors: BMI, age, postmenopausal status, smoking, physical activity, level of education, moderate alcohol consumption, n-3/Zn supplementation, total energy intakes and specific macronutrients intakes: carbohydrates, proteins, total fats, SFA, MUFA, n-6 PUFA, n-3 PUFA, TFA and cholesterol per day

* *p* < 0.05; regression coefficients are significant at the 0.05 level (2-tailed).

** *p* < 0.01; regression coefficients are significant at the 0.01 level (2-tailed).

PL, phospholipids; 18:0, stearic acid; 16:1n-7, palmitoleic acid; 20:3n-6, di-homo-gamma-linolenic; 20:5n-3, eicosapentaenoic acid; D9D, delta-9 desaturase (i.e., D9D-16; stearoyl-CoA desaturase 1, SCD-1); D6D, delta-6 desaturase; D5D, delta-5 desaturase; BMI, Body Mass Index; PUFA, polyunsaturated fatty acids; ANOVA, analysis of variance; *R*^2^, coefficient of determination; *β* (Beta), standardized linear regression coefficients; B, unstandardized linear regression coefficients; CI, confidence intervals; *p*, statistical significance. Individual FAs were expressed as a percentage of total identified FAs.

The proportions of 20:4n-6 were not predicted by BMI (data not shown), but were significantly predicated by the model which included the 18:2n-6 content and estimated activities of D6D and D5D, *R^2^* adjusted =1.000 (Supplementary Table S6).

### 3.6. Linear regression analyses for prediction of cardiometabolic risk indicators by selected FAs and estimated desaturases activities

In order to confirm the associations of FAs profiles and desaturase activities with indicators of cardiometabolic risk, we performed regression analyses in the opposite way: FAs profiles and desaturase activities were placed as predictors (with all other possible confounders, including age, postmenopausal status, smoking, physical activity, educational level, all dietary data and supplements use), while dependent variables were anthropometric, clinical and biochemical indicators of cardiometabolic risk. For biochemical indicators and BP, as predictors were included also anthropometric parameters.

Desaturase activities (particularly D9D and D6D) together with physical activity and age, significantly predicted anthropometric measures (BMI, FM, FM%, VFL, WC, HC, WHR and WHtR), while reported nutritional intakes, postmenopausal status, smoking and educational level did not have significant influence (Table 7). In contrast, the examined clinical and biochemical indicators of cardiometabolic risk were not predicted by desaturases activities, nor by selected FAs profiles, except for ALT (which was negatively predicted by 18:3n-3 and positively with age) and ratio ALT/AST (which was positively predicted by 20:5n-3, 16:1n-7 and VFL) (Supplementary Table S7). This remained significant also when subjects with n-3 PUFA supplementation were excluded from analyses (data not shown). All other biochemical data (as well as BP) were predicted mostly by anthropometric data (particularly measures of centripetal and visceral adiposity), less by age (Supplementary Table S7).

**Table 7:** Linear (stepwise) regression models for prediction of anthropometric indices by FAs and desaturase activities in erythrocytes PL.

Dependent variable	Model	Adjusted *R^2^*	ANOVA *p*	Coefficients	*β*	*B*	95% *CI* for *B*
							Lower bound upper bound
BMI	1	0.443	<0.001	(Constant)		1.239	[0.989, 1.490]**
				Physical activity	−0.476	−0.105	[−0.163, −0.047]**
				D9D	0.413	0.305	[0.112, 0.499]**
FM%	1	0.422	<0.001	(Constant)		1.473	[1.078, 1.869]**
				Physical activity	−0.558	−0.191	[−0.282, −0.100]**
				D9D	0.285	0.327	[0.021, 0.632]*
FM	1	0.420	<0.001	(Constant)		1.167	[0.540, 1.793]**
				Physical activity	−0.525	−0.284	[−0.429, −0.140]**
				D9D	0.331	0.599	[0.115, 1.083]*
VFL	1	0.625	<0.001	(Constant)		−1.832	[−2.997, −0.666]**
				Age	0.489	1.250	[0.638, 1.861]**
				Physical activity	−0.311	−0.293	[−0.504, −0.083]**
				D9D	0.280	0.883	[0.156, 1.611]*
Waist circumference	1	0.347	<0.001	(Constant)		1.755	[1.508, 2.001]**
				Physical activity	−0.390	−0.058	[−0.101, −0.014]*
				D6D	0.369	0.240	[0.049, 0.430]*
Hip circumference	1	0.344	<0.001	(Constant)		1.972	[1.847, 2.098]**
				Physical activity	−0.458	−0.047	[−0.076, −0.018]**
				D9D	0.334	0.114	[0.017, 0.210]*
WHR	1	0.082	0.050	(Constant)		−0.231	[−0.367, −0.094]**
				D6D	0.329	0.124	[0.000, 0.248]*
WHtR	1	0.349	<0.001	(Constant)		−0.452	[−0.708, −0.197]**
				Physical activity	−0.418	−0.064	[−0.109, −0.020]**
				D6D	0.352	0.239	[0.042, 0.437]*

MODEL 1– predictors: age, postmenopausal status, smoking, physical activity, level of education, moderate alcohol consumption, n-3/Zn supplementation, total energy intakes and specific macronutrients intakes: carbohydrates, proteins, total fats, SFA, MUFA, n-6 PUFA, n-3 PUFA, TFA and cholesterol per day, FAs composition and desaturase activities in erythrocytes PL.

* *p* < 0.05; regression coefficients are significant at the 0.05 level (2-tailed).

** *p* < 0.01; regression coefficients are significant at the 0.01 level (2-tailed).

BMI, Body Mass Index; FM%, Percentage of fat mass; FM, Fat mass; VFL, visceral fat level; WHR, waist to hip ratio; WHtR, waist to height ratio; D9D, delta-9 desaturase; D6D, delta-6 desaturase; ANOVA, analysis of variance; *R*^2^, coefficient of determination; β (Beta), standardized linear regression coefficients; B, unstandardized linear regression coefficients; CI, confidence intervals; *p*, statistical significance. Individual FAs were expressed as a percentage of total identified FAs.

### 3.7. Selected FAs and activities of desaturases across different BMI-categories

**Figure 1:**
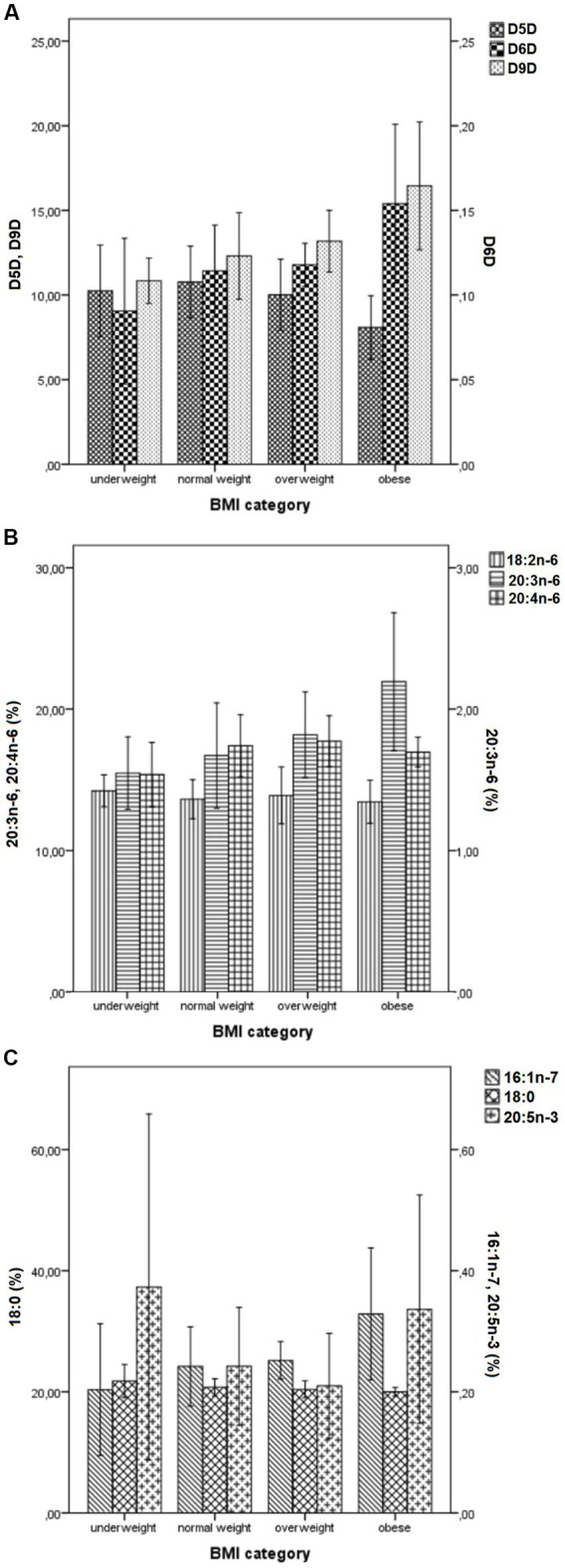
Mean values (± *SD*) of desaturase activities **(A)** and levels of selected FAs (18:0, 16:1n-7, 18:2n-6, 20:3n-6, 20:4n-6 and 20:5n-3) **(B,C)** across different BMI- categories. BMI, Body Mass Index; *SD*, standard deviation; Panel **(A)**: D9D, delta-9 desaturase activity (multiplied by 1,000); D6D, delta-6 desaturase activity; D5D, delta-5 desaturase activity; Panel **(B)**: 18:2n-6, linoleic acid; 20:3n-6, di-homo-gamma-linolenic; 20:4n-6, arachidonic acid; Panel **(C)**: 18:0, stearic acid; 16:1n-7, palmitoleic acid; 20:5n-3, eicosapentaenoic acid. Values represent mean ± *SD.* Individual FAs were expressed as a percentage of total identified FAs.

Figure 1 and Supplementary Table S8 show the means of selected FAs (18:0, 16:1n-7, 18:2n-6, 20:3n-6, 20:4n-6 and 20:5n-3) and desaturase activities across different BMI-categories. There was the inverse U-shaped (or the inverse J-shaped) association between D5D activity and BMI-categories (Figure 1A). Both underweight and overweight subjects had lower estimated D5D activities, compared with normal weight subjects, while obese subjects had the lowest D5D activities compared to all others (Figure 1A). The inverse U-shaped pattern was shown also for 20:4n-6, while the direct U-shaped pattern was shown for 20:5n-3 (Figures 1B,C). In contrast, levels of 16:1n-7, 20:3n-6, 18:0, as well D6D and D9D activities showed quite linear (or even exponential) change across BMI-categories (an increase for all except for 18:0, for which there was a decrease), while no change is seen for 18:2n-6 (Figures 1A–C). In Supplementary Table S8 are given ANOVA tables for statistical difference between different BMI-categories. The small numbers of underweight, overweight, and obese subjects did not allow detecting significant differences between different BMI-groups, except for the most extreme ones. For 16:1n-7, 20:3n-6, D6D and D9D activities, the most significant differences were between underweight and obese subjects, while for D5D activity, the most significant differences were between normal weight and obese subjects. There was no significant difference between the different BMI-groups regarding 18:0, 18:2n-6, 20:4n-6, and 20:5n-3 levels.

To overcome the problem with small numbers in some BMI categories, we performed the same analyses across different BMI-quartiles. The results were quite the same, with linear/exponential increases in D9D and D6D activity, and the inverse U-shaped (or the inverse J-shaped) change in D5D activity across BMI-quartiles (Supplementary Figures S2, S3; Supplementary Table S9). Quite similar figures and patterns were obtained if FM, FM% or VFL quartiles were used (data not shown).

In order to further explore the inverse U-shaped change in D5D activity across BMI-categories/quartiles, we applied the non-parametric locally weighted regression and stratified linear regressions across BMI quartiles. The locally weighted smoothed regression line with the Epanechnikov kernel showed that there was an increasing trend for D5D activity up to BMI of about 23–24 kg/m^2^, and after there was a decreasing trend (Supplementary Figure S3A). Stratified linear regressions lines across BMI quartiles have given very similar results and have shown that up to 3rd quartile (BMI 22–25), the regression lines have shown positive association of with BMI, then in the 3rd quartile it plateaued (no association), and after, in the 4th quartile (BMI >25), the negative association with BMI was shown (Supplementary Figure S3B). However, only in the 4th quartile (which included only overweight and obese subjects), there was a significant negative association, *R*^2^ = 0.297 (Supplementary Figure S3B). The later was confirmed by stratified linear regression models: only in the 4th quartile there was a significant negative effect of BMI on D5D activity in both univariate (when only BMI was used as predictor) and multivariate (with all predictors included) stratified regression models (Supplementary Table S10).

## 4. Discussion

This study showed that product/precursor estimated desaturase activities of D9D, D6D and D5D, and the FAs profiles of 18:0, 16:1n-7, 20:3n-6 and 20:5n-3 in erythrocyte membranes PL are significantly associated with the examined indicators of cardiometabolic risk among non-diabetic otherwise healthy Serbian women. The positive association of desaturase activities with adiposity was crucial and explained the association with other (biochemical and clinical) markers of cardiometabolic risk (together with age), since in our study we did not find age- and adiposity- independent associations of the mentioned FAs levels and desaturase activities with the levels of lipids, GLU, transaminases or BP (except for the associations of 20:5n-3 with ALT/AST ratio). Body mass index (together with age and smoking) was a significant predictor for almost all of the above-mentioned FAs (except 20:5n-3) and desaturases, and explained up to 25% of their variance. Vice versa, the activities of desaturases (particularly D9D and D6D) were significant predictors of anthropometric indices, together with age and physical activity, while (interestingly) dietary factors, postmenopausal status, smoking and educational level did not have significant influence in multiple regression models.

Our results on the associations of desaturase activities with the level of adiposity agree with almost all published data (20, 22, 27), even in children (25, 129, 130), but disagree with some studies concerning the adiposity-independent associations with clinical and biochemical biomarkers of cardiometabolic risk. For example, our results for adiposity-mediated D6D and D9D associations with metabolic profile are in agreement with a prospective study in elderly men (32), but disagree with D5D associations, since in that study the inverse association of D5D with adverse metabolic profile was independent from BMI. In a 10-year longitudinal study in Chinese women and men, higher serum D9D-18 activity, 16:1n-7, 18:1n-9, 20:3n-6 levels and lower D5D activity at baseline were predictors of development of metabolic abnormalities and transition of MHO to metabolically unhealthy obesity (MUO) phenotype, independently of sex, age, and BMI (97, 98). Nevertheless, in metabolically healthy normal-weight subjects who progressed to metabolically unhealthy normal-weight phenotype, these differences in desaturase activities were not observed (97). Similar results were obtained by the same authors in additional cross-sectional studies, where subjects with MUO had higher serum 16:1n-7, 18:1n-9, 20:3n-6 levels, higher D9D-16/D9D-18 activities and lower D5D activity, compared with subjects with MHO, despite similar BMI (97, 98). Similarly, serum ALT was independently positively associated with D9D activity in elderly men in one study (99). Risk for hypertension was associated with 16:0, 16:1n–7, 22:6n–3, 20:3n–6, D9D, D6D and D5D activity among Chinese cohort, independently of age, gender, BMI and other confounders (131). Possible reasons for discrepancy in these results with ours could be the differences in the studied populations and the analyzed FAs pools. In our study healthy women were studied, majority of them were of younger age, normal weight and only several of them had MetS. If the subjects with older age, higher level of adiposity and more adverse metabolic profile were included, or men (who are, e.g., more predisposed to NAFLD) (132), probably we would see also the adiposity-independent effects. Additionally, the ethnic differences may also contribute to the observed discrepancy in the results (57, 103). Apart from possible genetic differences between ethnic populations in *Scd1*, *Fads2* and *Fads1* polymorphisms (105, 133, 134), also dietary differences between ethnic populations may contribute (103). For example, in Caucasians on a high carbohydrate diet, 44% of variance in serum TAG was explained by D9D activity, while on high fat diet, only 11% of variance in serum TAG was explained by D9D activity (135). In our study, a high-fat/low-carbohydrate dietary pattern was identified, so the associations between desaturase activities and serum TAG levels may be less observable. Therefore, many differences between studied populations can contribute to contradictory results. Nevertheless, according to here presented data, in healthy, non-diabetic, predominantly younger and non-obese Serbian women, the connection of FAs profiles and desaturases activity with adverse metabolic profile was mostly conveyed by the effect of increased adiposity.

The association of adiposity and desaturase activities can be bidirectional: the increased adiposity produced by increased energy intake can change the expression and activities of desaturases in various tissues, and vice versa, the activities of desaturases may contribute to increased adiposity.

In support of the effect of adiposity on desaturases activities, weight loss in humans achieved by various dietary caloric reductions (with or without exercise) or metabolic surgery, significantly increased the D5D activity, while decreased the D6D and D9D activities and 16:1n-7, 20:3n-6, 18:3n-6 and 18:0 levels in erythrocyte membranes, whole plasma lipids, or separate plasma/serum lipid fractions (97, 98, 136–140). The reasons for the differences in the effect of weight loss on 18:0 in the cited studies compared with this study findings are unclear.

Likewise, animal studies with diet induced obesity (DIO) are in line with findings in humans, even though there can be some differences and discrepancies. For example, in the liver of mice on high-fat diets (HFD) or high sucrose diets (HSD), the increased, decreased or unchanged mRNA and protein expressions and estimated desaturases activities were shown (94, 141–146). The reasons for these discrepancies in animal results are not clear, and can be results of different experimental designs, animal strains, mode of expressing desaturase activity, type of diet or level of adiposity achieved.

The expression and activity of desaturases is controlled by many factors, including nuclear receptors: sterol regulatory element binding protein-1c (SREBP1c), liver X receptors (LXR), retionoid X receptor (*r*XR), carbohydrate regulatory element binding protein/ Max-like factor-X (ChREBP/MLX), PPAR-gamma peroxisome proliferator activated receptor (PPAR) alpha and gamma, and many others (64, 147, 148). In addition, epigenetic alterations (i.e., methylation of DNA), stability and degradation of the enzyme protein, as well as changes in the cytosolic NAD+/NADH ratio may contribute to changes in desaturases activities. In general, desaturases expressions and activities are stimulated by insulin, carbohydrates, and inhibited by PUFA, while the effects of leptin, SFA and MUFA can vary, depending on specific desaturase (64, 147–150).

Even though bio-mechanisms by which increased adiposity affects desaturase activities are still not well defined and can involve various pathways, it seems that the desaturase activity regulation by insulin and leptin can provide satisfying explanations.

Increased adiposity causes insulin resistance and leptin resistance. Insulin is one of the major stimulators of desaturase activities in many tissues, through induction of SREBP-1c, a nuclear factor which regulates lipogenesis and increases all three desaturases’ expressions and activities (151). Insulin increases the SREBP-1c gene transcription and mRNA stability, proteolytic maturation and nuclear transition of SREBP-1c protein, and inhibits its proteasomal degradation. Even though in obesity there is insulin resistance in many tissues for various actions of insulin, interestingly, there is no resistance of the effect of insulin on SREPB-1c induction in the liver (152, 153). Consequently, the compensatory increased insulin levels in insulin resistant states (including obesity) stimulate the SREPB-1c and increase expression and activity of all three desaturases, which was confirmed by studies in streptozocin induced diabetic animals (147, 154). However, it remains unclear which mechanism is responsible for different regulation of D5D by insulin in obesity, compared with D9D and D6D. Most studies show that D9D, D5D and D6D are regulated by the same factors (including insulin) in the same direction (64, 147). However, it seems that there is a differential effect of insulin on desaturases, depending on desaturase itself, the concentration of insulin and insulin sensitivity (155). Some studies suggested that regulation by insulin can be differential for D5D compared with D6D and D9D (156–158). For example, in streptozocin induced diabetes, the inhibition of D6D is much stronger than inhibition of D5D, indicating that D6D more strongly depends on insulin than D5D (156). Another study in animals showed no effect of insulin on the estimated D5D activity in hepatocytes or myocytes (159). Moreover, while in small, physiological doses insulin can have an increasing effect on the decreased D5D activity in insulin deficient diabetic rats or humans with type 1 diabetes (156, 160, 161), it was shown, in contrast, that in non-diabetic rats, insulin in higher doses inhibits the D5D activity in hepatocytes, measured by the labeled FAs incorporation (162). Unfortunately, we did not measure the insulin levels in our subjects, and there are no studies on the effect of insulin in non-diabetic subjects, to test the hypothesis of the biphasic effect of insulin on D5D activity (e.g., kinetic studies involving hyperinsulinemic-euglycemic-clamp and measuring desaturase activities in the blood by the use of stable isotopes).

With increased adiposity there is also leptin resistance. Leptin is known as potent inhibitor of D9D and D6D transcription and activation, mostly through its indirect central effects in the brain, but also some direct effects of leptin on the liver and adipose tissue can exist (163). In leptin deficient *ob/ob* animals, leptin administration centrally (i.e., intra-cerebroventricularly) decreased liver and adipose tissue *Scd1* and *Fads2* expression (unfortunately, *Fads1* expression was not examined), through its effects on SREBP-1c, ChREBP, PPAR-gamma, PPAR-alpha, PGC-1alpha, AMP-activated protein kinase (AMPK), STAT3, and ERK1/2 MAPK pathway (163–166). Apart from influencing mRNA expression, leptin also decreased D9D half-life, which could additionally contribute to decreased D9D activity (147). Nevertheless, with increased adiposity, there is a central and peripheral resistance on leptin effects (caused by its diminished transport across the blood–brain barrier, decrease in the leptin receptor mRNA expression and density in the hypothalamus, hepatocytes, adipose tissue and muscles, as well as the deterioration of leptin-receptor function and signaling) (167), and therefore leptin cannot exert its inhibitory effect on *Scd1* and *Fads2* expression. On the other hand, to the best of our knowledge, no studies examined effect of leptin on *Fads1* expression and D5D activity, even though in the mentioned study with central leptin infusion (163), also the increased liver 20:4n-6 values were shown, in parallel with increased 18:2n-6 values, which could indicate inhibited D6D activity, but stimulated D5D activity with leptin administration. All together, these data could suggest that in leptin resistant states (including simple obesity), there could be lack of both inhibitory effect of leptin on D6D and D9D activity and stimulatory effect of leptin on D5D activity.

Nevertheless, as already mentioned, the association of the increased adiposity and desaturase activities can be bidirectional. Vice versa, the increased activities of desaturases can predispose for adiposity development. For example, mice that lack the *Scd1* gen activity are lean, resistant to DIO (despite increased food intake), have increased basal metabolism and fat oxidation, decreased *de novo* lipogenesis (DNL) and fat accumulation in the liver and adipose tissues, lower insulin resistance and more favorable metabolic profile (lower blood TG, GLU and insulin levels). On the other hand, they have increased oxidative and inflammatory liver damage and endoplasmic reticulum stress, due to increased SFA accumulation and oxidation in hepatocytes, as well as increased atherosclerosis (168–172). Liver-specific knock-out of *Scd1* gen protects from the HSD induced adiposity in the same degree as the whole body *Scd1* knock-out, but not from the HFD induced adiposity, indicating that in rodents, the liver D9D has a vital role for obesity development particularly in circumstances with high carbohydrate intakes, while in circumstances with high fat intakes, the adipose tissue D9D is more involved (171, 173). Especially liver D9D-18 activity (but not D9D-16 activity) contributes to DIO development, as shown in transgenic animals on HSD (174). Pharmacological inhibition of D9D showed similar effects as the genetic knock-out of *Scd1* (169). The similar (but not identical) effects were also was shown with knock-out of *Fads1* and *Fads2* genes (175–181) or pharmacological inhibition of D5D and D6D in mice (94, 145, 182): protection from HFD- DIO, insulin resistance, unfavorable metabolic and pro-inflammatory profiles in the case of D5D deficiency, but only protection from DIO with partial protection from unfavorable metabolic profiles in case of D6D deficiency. In accordance, even in humans genetic polymorphisms of *Scd1* (183–188), *Fads2* and *Fads1* (189–202) genes were related to increased predisposition to weight gain, increased adiposity, abdominal adiposity and related traits (insulin resistance, high GLU, TAG and LDL-C, low HDL-C, and pro-inflammatory adipokine/cytokine/chemokine profiles), with the effects varying depending on dietary, gender and ethnic interactions (102, 184, 185, 187, 188, 194, 197, 203–205) Therefore, the evidence supports both directions in the associations of increased adiposity with desaturases activities.

The more significant associations of D6D and D5D with increased adiposity compared with D5D found in this study can be causal (as explained above), but could also reflect the methodological issues in estimation of enzymatic activity. Desaturase indices, calculated as product/substrate ratios, are only an indirect measure of desaturase activity and do not have to represent the real activity of the enzyme (206). For example, there could be also a process of retro-conversion of 20:4n-6 to 20:3n-6 (207). Moreover, the product of D5D, 20:4n-6, is further metabolized to 22:4n-6 by Elov2, so the activity of this elongase could additionally affect the estimated D5D activity. In our study, 22:4n-6 values were about 25% of 20:4n-6 values, which indicates that significant proportion of 20:4n-6 is metabolized to 22:4n-6. However, in our study we did not find the associations of the estimated Elov2 activity (calculated as 22:4n-6/20:4n-6 or 22:5n-3/20:5n-3 ratios) with anthropometric indices, nor with D5D activity (data not shown), so the effect of Elov2 did not significantly influence results for D5D, which is in line with the literature data (97). Similarly, membrane bond 20:4n-6 in erythrocytes is known to be non-enzymatically oxidized/peroxidized or metabolized to eicosanoids and endocannabinoids by activity of phospholipases, cyclooxygenase, lipoxygenase, cytochrome P450-dependent enzymes, phosphatases and diacylglycerol-lipase, and their activity can be changed in obesity and MetS, which are considered as pro-inflammatory states (208, 209). Unfortunately, the eicosanoid and endocannabinoid levels in the blood were not assessed in this study. Furthermore, while the products of D6D and D9D (i.e., 20:3n-6 and 16:1n-7) are rarely found in the diet, and mostly reflect endogenous synthesis, 20:4n-6 is also provided by animal dietary sources (including meat, poultry, eggs, fish, and dairy products) (210). Therefore, its proportions in erythrocytes PL can be increased by dietary intakes and can erroneously inflate the estimated D5D activity with the increased dietary consumption of animal sources food. Nevertheless, it still seems that 20:4n-6 much more derives from endogenous conversion of 18:2n-6, 18:3n-6 and 20:3n-6 (211, 212), since large dietary intakes of 18:2n-6 much overcome low dietary intakes of 20:4n-6, and it is estimated that dietary 20:4n-6 contribution is less than 1% of total 20:4n-6 pool (213). In agreement, some studies showed only slightly lower 20:4n-6 levels in vegans compared with omnivores, while others even did not find the difference, despite huge differences in 20:4n-6 intakes (214, 215). In our study, we could not distinguish the intake of 20:4n-6 from different sources (animal vs. plant), and thus endogenous from exogenous 20:4n-6, due to methodological limitations, but 20:4n-6 levels were positively predicted by the levels of 18:2n-6, D6D and D5D, and that model completely explained the 20:4n-6 content, indicating that 20:4n-6 levels are principally determined by endogenous synthesis in our study. Interestingly, both D6D and D5D desaturases equally explained the variability in 20:4n-6 (together up to 71.6% of 20:4n-6 variability in our model). The addition of 18:2n-6 in the model explained 100% of variability of 20:4n-6. Finally, our data could also suggest that D5D activity could be more influenced by some intrinsic factors (e.g., genetic polymorphism) than by external factors (compared with D6D and D9D), which is in agreement with genetic polymorphism studies that underline genetic influence on D5D activity (216–219). For example, in a study which examined the FAs profiles in weight-discordant monozygotic twin pairs, the estimated activity of D5D was not different with obesity, while the estimated activities of D6D and D6D were increased in the obese twin (220). Nevertheless, the results of genetic studies on *Fads1* polymorphisms and obesity are often contradictory, with more effects observed in children compared with adolescents and adults (192, 193, 198, 202), and it appears that other factors can still overcome the genetic influences.

Despite all these considerations, it seems that the most reasonable explanation for the less significant associations of increased adiposity with D5D activity is that relation of D5D activity with anthropometric indices was the inverse U-shaped. Both underweight and overweight subjects had lower estimated D5D activities, compared with normal weight subjects in our study, while obese subjects had the lowest D5D activities compared to all others. The non-parametric locally weighted regression showed an increasing trend for D5D activity up to BMI cut-off of about 23–24 kg/m^2^, and after which there was a decreasing trend. Nevertheless, in the stratified analyses across BMI quartiles, only in overweight/obese subjects there was a significant association of BMI with D5D activity. However, due to the small number of subjects in quartiles, we could not perform proper stratified regression analyses, and these results should be interpreted with caution. In contrast, D6D and D9D activities (as well as 16:1n-7 and 20:3n-6 levels) showed a more linear (exponential) increase from underweight to overweight subjects, while for the 18:0 content, a linear decrease was shown. These findings can be explained by stimulation of D6D and D5D by insulin (which directly correlates with anthropometric indices, i.e., level of adiposity), and the biphasic effect of insulin on D5D activity. While in the insulin sensitive subjects (with lower BMI) insulin can have positive effect on D5D, in the insulin resistant subjects (with higher BMI), there can be an inhibitory effect, as suggested by studies in rats (156, 160–162). Unfortunately, we did not asses the insulin levels in our subjects to estimate insulin sensitivity at least by HOMA-IR index (221). Additionally, measuring leptin levels could be of interest, to assess the effect of both low and high leptin levels on D5D activity. As mentioned above, there are not published studies on the effect of leptin on D5D activity (only on the effect on D6D and D9D activity) (163).

The inverse U-shaped association was un unexpected finding, and to the best of our knowledge, there are no studies which reported such association. In the majority of the published studies, only normal weight and obese subjects were compared, and rarely the subjects with lower BMI were involved in research of FAs profiles. Our unexpected finding raises the need for more studies in this group of subjects, but interestingly, there are no studies on that topic. Additionally, subjects with eating disorders or subjects with lipodystrophic syndromes can be studied. Of note, the later ones represent insulin resistant subjects (222), while in eating disorders – e.g., anorexia nervosa (AN), both increased and diminished insulin sensitivity can be seen, depending on the muscle mass atrophy and abdominal fat accumulation (223, 224). Lipodystrophic subjects have disturbed lipid cell metabolism and they could have genetically determined changes in desaturases activities (225, 226), while subjects with AN can have completely different nutrient intakes (particularly of essential FAs) and disturbed metabolism and endocrine function (227–232), thus they make specific groups of underweight subjects, not suitable for comparison with general population.

As mentioned above, in our study three FAs were linearly correlated with anthropometric indices of adiposity, and both centripetal and peripheral fat accumulation: 20:3n-6, 16:1n-7 and 18:0. However, 18:0 correlated less with adiposity indices than 20:3n-6 and 16:1n-7. Our findings for 20:3n-6 and 16:1n-7 are in accordance with all other findings in the literature, but our data for 18:0 can differ from other studies, where the levels of 18:0 in both erythrocytes PL and plasma (whole plasma, plasma FFA, CE and PL) were positively related to indices of adiposity (20, 26, 98, 101, 233–235). However, more obese subjects and both men and women were included in the mentioned studies, and there could be also some dietary influences.

With increased adiposity, the levels of 18:0 could be decreased because of the increased activity of D9D (which transforms 18:0 to 18:1n-9) and the decreased activity of Elov6 (which produces 18:0 from 16:0) (97, 220). In our study, we also observed a significant negative correlation of the estimated Elov6 activity (calculated as 16:1n-7/18:1n-9 and 16:1n-7/18:1n-7 ratios) with the indices of adiposity and visceral fat accumulation (data not shown). This is in accordance with the mentioned study in weight discordant twins (220) and study of Zhao and associates (97).

Another interesting question arises from our research: it is known that n-3 PUFA can ameliorate NAFLD, ALT and AST levels (236–239), but here we have found a positive correlation of 20:5n-3 with the ALT/AST ratio, as an indicator of liver steatosis, even after controlling for all possible confounders (including dietary n-3 PUFA intakes and n-3 PUFA supplements usage), or after excluding the subjects with n-3 PUFA supplementation. This is the opposite from what one would expect, considering that people with NAFLD have the lower n-3 PUFA content in their livers and that n-3 PUFA can ameliorate NAFLD, by increasing beta oxidation and decreasing DNL and liver TAG synthesis (146, 236, 240–243). One possible explanation is that in NAFLD there can be a lower conversion rate of 20:5n-3 to 22:6n-3. Indeed, we observed in this study a significant negative correlation of estimated “putative D4D” activity (calculated as ratio 22:6n-3/20:5n-3) with ALT/AST ratio (*r* = −0.536, *p* < 0.001) and ALT (*r* = −0.353, *p* = 0.002) (unpublished observations). The linear regression models also confirmed the negative association of “putative D4D” activity with ALT/AST ratio (data not shown). Even though instead of putative D4D enzyme there are actually several different steps during process of conversion of 20:5n-3 to 22:6n-3, which include Elov2/5, D6D and peroxisomal beta-oxidation by acyl-CoA oxidase and 17β-hydroxysteroid dehydrogenase 4 (64, 146), the ratio 22:6n-3/20:5n-3 can show the rate of this conversion, which in humans is quite low (240), and can be further reduced in states of hepatic steatosis and steatohepatitis (244–246), so there could be accumulation of 20:5n-3 and 22:5n-3 in such states. In agreement, also 22:5n-3, the intermediate Elov2/5 product during this conversion, positively correlated with ALT/AST ratio, although less than 20:5n-3. In addition, we also observed the positive correlation of 22:5n-3 with GGT (*r* = 0.469, *p* = 0.012). However, there could be other possible mechanisms to explain the positive association of 20:5n-3 with ALT/AST ratio. For example, there could be a possibility of increased desaturation and elongation of 18:3n-3, increased retroconversion of 22:6n-3 to 20:5n-3, reduced beta-oxidation of 20:5n-3 or reduced transformation by cyclooxygenase and lipoxygenase to eicosanoids in hepatic steatosis. However, quite the opposite was described in NAFLD (142, 239, 240, 243, 247), and both the retroconversion of 22:6n-3 to 20:5n-3 and conversion of 18:3n-3 to 20:5n-3 and 22:6n-3 in humans are relatively inefficient (207, 248–251). Furthermore, ALT is more a marker of liver steatosis and gluconeogenesis, while AST is more a marker of cellular damage, and it could be possible that the anti-inflammatory and anti-oxidative 20:5n-3 properties overcome its influence on hepatic lipid accumulation (243, 252, 253). In agreement, supplementation with n-3 FAs (fish oil) increased ALT/AST ratio in rats on a hypercholesterolemic HFD (254), and in some studies in humans, the more potent effect on AST than ALT reduction was observed, but not in all, and in some even more potent effect on ALT reduction was shown (236, 240, 243, 255, 256). Therefore, the observed positive association probably cannot be explained by the possible effect of 20:5n-3 on ALT/AST ratio. Moreover, none of our subjects had severe liver damage, and the association was present across different BMI-categories and BMI-quartiles, although the association was stronger in higher BMI quartiles (data not shown).

The positive associations of 16:1n-7 and VFL with ALT/AST ratio is not surprising, bearing in mind that 16:1n-7 is the one of the best indicators of the increased DNL, which is a hallmark of NAFLD (77, 240). Even though many other FAs are also the products of DNL, including SFA from C12:0 to C18:0 and other MUFA (18:1n-9 and 18:1n-7) (42, 48), 16:1n-7 is probably the best indicator of DNL, due to its low abundance in common dietary sources and high correlation of D9D activity with activity of other enzymes involved in DNL, since they are all regulated by the same regulatory elements, SREBP-1c and ChREBP (77, 257, 258). In accordance, D9D-16 is much better indicator of DNL compared with D9D-18 or ratio 16:0/18:2n-6 (also known as DNLIndex) (40, 77). Our data also confirm the association of D9D with ALT and ALT/AST ratio, and very strong association of D9D with VFL.

The found associations of indicators of cardiometabolic risk as well as desaturase activities and FAs profiles with age, postmenopausal status, smoking, moderate alcohol consumption, physical activity and educational level are more/less in accordance with previously published data (20, 53, 54, 59, 259). Smoking, age, and physical activity all increase oxidative stress, which can affect desaturase activities (74, 95, 96, 260, 261). Age was associated with 16:1n-7 and D9D, because of age-related increases in visceral fat mass (262–264). When VFL was also included in the model, VFL became the only significant predictor of 16:1n-7 and D9D (data not shown). Data on menopausal status are scarce, and one study did not find the differences in FAs profiles (106). However, some other studies have shown some differences (61), but the study pre- and post-menopausal groups were very different regarding age and BMI, so the effect of menopause could not be clearly distinguished from the effects of age and BMI. In one large-sample study (265), only the levels of 16:0 were negatively associated with menopause, which in accordance with our study results. In our study, after adjusting for multiple confounders (including age), only the negative association of 16:0 with menopause remained significant (*r_pb_* = 0.256, *p* = 0.033). According to literature data, smoking has negative associations with D5D (and much less D6D) activity, while has positive associations with D9D activity (20, 74, 76, 96, 266–268). In our study we also have found negative associations with D5D and positive associations with D9D activity, but after adjustments for multiple confounders only negative associations with D5D activity remained. In majority of the studies, physical activity was in positive association with D5D activity, and negative association with D9D and D6D activities (269–273), what is similar with our results, even though in linear regression models the physical activity level was not a significant predictor, indicating that other factors (e.g., BMI, smoking, age) confounded this association, which is in agreement with results of Warensjö and associates (20).

Surprisingly, in our study we did not obtain the significant associations of both desaturase activities and biochemical indices with dietary factors, except for negative associations of anthropometric indices with total energy and macronutrients intakes (except for n-3 PUFA), negative associations of PUFA intakes (particularly n-6 PUFA) with biochemical indices of cardiometabolic risk, and positive associations of 20:5n-3 with n-3 PUFA intake and with n-3 PUFA or n-3 PUFA/Zn supplementation. Nevertheless, only the associations of 20:5n-3 with n-3 PUFA intake and n-3 PUFA and Zn supplementation did remain significant in linear regression models. The associations of total n-3 PUFA dietary intakes (from both plant and animal sources) with 20:5n-3 levels, but not 22:6n-3 levels, can be explained by low conversion rates of 18:3n-3 to 22:6n-3 (much lower than 20:5n-3) (215, 250, 274). In contrast to what could be expected from the literature (51, 64, 65, 275), no other significant associations of desaturase activities or FAs levels were found with dietary data obtained from 24 h-recalls, related to total energy, carbohydrate, protein, total fat, and (particularly) SFA, MUFA and PUFA intakes. One of the reasons for that is that only two 24 h-recalls were used for dietary assessments, while usually much longer dietary surveys (up to 15 days), or validated FFQ were used to precisely estimate different dietary fats intakes (210, 276, 277). We did not have FFQ data (only FPQ) to estimate dietary intakes over a year, and 24 h-recalls can reflect recent dietary intakes, while erythrocytes FA profiles more represent the long-term dietary intakes compared with plasma FA profiles (67, 278, 279). Nevertheless, also other authors noted a week correlation between macronutrient nutritional intakes and desaturase activities in erythrocytes, except for n-3 PUFA (22:6n-3, 20:5n-3) and TFA (278). There is also the problem with self-reported data, including under-reporting, which is particularly common among obese subjects (109, 280). In line with that, the possible under-reporting could also explain the observed negative associations of total energy intake and intakes of almost all macronutrients with anthropometric indices indicative for obesity.

### 4.1. Strengths and limitations

The strengths of this study are in a very thorough analysis and controlling for many possible confounders. Additionally, we analyzed FA composition in erythrocyte PL (separated by TLC), which more reflects the long-term dietary intakes and endogenous metabolism in the liver, compared with other sources, and is less susceptible to confounding in estimation in hyperlipidemic states (90, 92). The major study limitation is the relatively small sample size. We had made the study power calculation, and even though we here obtained bit lower correlation coefficients (the lowest *r* = 0.234), our study-power is above 80% for majority of the obtained correlations (all above *r* = 0.316). Nevertheless, the small number of participants did not allow adequate statistical power for multiple adjustments implemented (281). We performed the Bonferroni correction to avoid an increased risk of a type I error when making multiple statistical tests, but this probably underestimated the influence of possible confounding factors with lower correlation coefficients than *r* = 0.450, which can increase the risk of a type II error (281). Moreover, the number of involved subjects was not sufficient enough to make proper stratification analyses. Because of that, our exploratory findings need to be confirmed in a larger cohort, particularly for further stratification analyses. Another study limitation is that in our cohort, the number of underweight, overweight and obese subjects was significantly lower than the number of normal-weight subjects, and half of the subjects were younger than 30 years. Therefore, our results probably mostly relate to younger and normal-weight women. Our study included only Serbian women, and the results cannot be directly extrapolated to men (or women of different race/ethnicity). Hence, our findings need to be confirmed in other populations, with probably larger samples included, and more equally represented age and BMI-categories. Further limitation is that the product/predictor estimation of desaturase activities was used to assess enzymatic activity, which does not have to reflect the real enzymatic activity (78, 79). Moreover, we did not assess the absolute concentration of FAs in erythrocyte PL, which would be ideal. However, the vast majority of the studies cited here also expressed their results as percentage of the total FA content (relative %), and there is a good correlation with the absolute concentrations in erythrocyte PL (101). Also, our chromatographic conditions were not tested for the separation of positional isomers of unsaturated FAs (e.g., two *cis* positional isomers for 16:1, 16:1n-6c and 16:1n-9c), as well as of their geometrical *trans* isomers. Nevertheless, our peak retention times were defined by a certified calibration standard mixture of *cis*-isomers retention times, and in erythrocyte PL the total content of the *trans* isomers is relatively low (on average, less than 2%) (101, 278, 282, 283). Moreover, our chromatographic conditions were not tested for the separation of FAME from dimethylacetal (DMA) and alk-1-enyl methyl ether (AME) derivatives, which are formed after basic/acidic transesterification of plasmalogen fraction of PL, contacting alk-1-enyl (vinyl) ether bonds (284–286). Furthermore, to assess the level of adiposity, the gold standards are dual-energy X-ray absorptiometry (DXA) and magnetic resonance/computed tomography (MRI/CT), while we in our study used the more applicable surrogate measures, which have their own limitations (287, 288). Nonetheless, our results were consistent across different indicators of adiposity, suggesting that our results are robust. In addition, our study would be more complete if we had measured the levels of insulin, leptin, certain eicosanoids and endocannabinoids, which would give more insights into pathophysiologic mechanisms. Nevertheless, the cross-sectional study design still does not allow making conclusions on the causality. Finally, as we already mentioned in the paragraph above, there are certain limitations of the performed dietary surveys.

## 5. Conclusion

This study confirmed that the levels of certain FAs (16:1n-7, 20:3n-6, and 18:0) and estimated desaturase activities in erythrocytes PL were significantly associated with anthropometric, clinical and biochemical cardiometabolic risk indicators in healthy, non-diabetic Serbian women. However, the associations of clinical and biochemical indicators of cardiometabolic risk were not independent from the associations with the level of adiposity. After controlling for multiple confounding factors (including age, postmenopausal status, smoking, physical activity, educational level, alcohol consumption, use of supplements and dietary macronutrient intakes), the level of adiposity was the most significant predictor of the estimated desaturase activities and 16:1n-7, 20:3n-6, and 18:0 levels, and mediated their association with biochemical and clinical indicators. Vice versa, desaturase activities predicted the level of adiposity, but not other components of cardiometabolic risk (if the level of adiposity was also accounted). While the associations of 16:1n-7, 20:3n-6, 18:0, D9D and D6D activities with anthropometric indices were linear (positive with 16:1n-7, 20:3n-6, D9D and D6D, and negative with 18:0), the associations of D5D activity with anthropometric indices were the inverse U-shaped. The only adiposity-independent association of FAs profiles with indicators of cardiometabolic risk was the association of 20:5n-3 with ALT/AST ratio, which requires further exploration. More studies are needed to explore the mechanisms of the observed associations of FAs profiles and desaturase activities with anthropometric, clinical, and biochemical cardiometabolic risk indicators, including larger-scale confirmatory studies.

## Data availability statement

The raw data supporting the conclusions of this article will be made available by the authors, without undue reservation.

## Ethics statement

The studies involving human participants were reviewed and approved by Institute of Occupational Health Niš Ethics Board. The patients/participants provided their written informed consent to participate in this study.

## Author contributions

IŠ wrote the manuscript and performed anthropometric measurements, nutritional interviews, software-exploration of nutritional data, and all statistical analyses. JDM, MT, NKV, TP, and JJ performed chromatographic and biochemical analyses. VS, JM, and MZ were involved in anthropometric and dietary assessment. All the authors contributed to the manuscript writing.

## Funding

The authors acknowledge financial support from the Ministry of Education, Science and Technological Development of the Republic of Serbia (project number III41030 2011-2017 and contract number: 451-03-68/2022-14/200015) and Ministry of Science, Technological Development and Innovation of the Republic of Serbia (contract number: 451-03-47/2023-01/200015).

## Conflict of interest

The authors declare that the research was conducted in the absence of any commercial or financial relationships that could be construed as a potential conflict of interest.

## Publisher’s note

All claims expressed in this article are solely those of the authors and do not necessarily represent those of their affiliated organizations, or those of the publisher, the editors and the reviewers. Any product that may be evaluated in this article, or claim that may be made by its manufacturer, is not guaranteed or endorsed by the publisher.
