# Supplementary material for: Associations of fatty acids composition and estimated desaturase activities in erythrocyte phospholipids with biochemical and clinical indicators of cardiometabolic risk in non-diabetic Serbian women: the role of level of adiposity
Source: Front Nutr. 2023 Jul 20;10:1065578. doi: 10.3389/fnut.2023.1065578 (PMC10397414; doi:10.3389/fnut.2023.1065578)
Supplement: Supplementary file 1 [file Data_Sheet_1.doc]

Supplementary Material

## Supplementary Tables

**Supplementary Table S1. Correlation of general and anthropometric characteristics with biochemical and clinical (BP) data (*N* =76)**

|  | **Age** | **Post-menop.** **^§^** | **Smoking^§^** | **Physical activity** | **Education** | **Alcohol^§^** | **n-3 suppl.** **^§^** | **n-3/Zn suppl.** | **GLU** | **TAG** | **HDL-C** | **LDL-C** | **TAG/**  **HDL-C** | **ALT** | **AST** | **ALT/AST** | **SBP** | **DBP** |
| --- | --- | --- | --- | --- | --- | --- | --- | --- | --- | --- | --- | --- | --- | --- | --- | --- | --- | --- |
|  | *r* | | | | | | | | | | | | | | | | | |
| **Age** | - | 0.707^**^ | 0.502^**^ | -0.323^**^ | -0.414^**^ | -0.235^*^ | 0.050 | 0.112 | 0.470^**^ | 0.526^**^ | -0.185 | 0.646^**^ | 0.465^**^ | 0.510^**^ | 0.122 | 0.519^**^ | -0.210 | 0.156 |
| **Post-menopause**^§^ | 0.707^**^ | - | 0.390^**^ | -0.110 | -0.317^**^ | -0.216 | -0.069 | -0.069 | 0.375^**^ | 0.325^**^ | -0.042 | 0.484^**^ | 0.265^*^ | 0.291^*^ | -0.014 | 0.202 | -0.072 | 0.039 |
| **Smoking**^§^ | 0.502^**^ | 0.390^**^ | - | -0.037 | -0.355^**^ | -0.195 | 0.138 | 0.191 | 0.177 | 0.342^**^ | -0.146 | 0.487^**^ | 0.310^**^ | 0.297^*^ | -0.055 | 0.325^**^ | -0.285^*^ | 0.066 |
| **Physical activity** | -0.323^**^ | -0.110 | -0.037 | - | 0.067 | -0.072 | -0.043 | -0.164 | -0.438^**^ | -0.309^**^ | 0.086 | -0.218 | -0.266^*^ | 0.028 | 0.117 | -0.200 | -0.260^*^ | -0.227 |
| **Education** | -0.414^**^ | -0.317^**^ | -0.355^**^ | 0.067 | - | 0.113 | 0.091 | 0.077 | -0.285^*^ | -0.277^*^ | 0.172 | -0.315^**^ | -0.268^*^ | -0.154 | 0.092 | -0.264^*^ | 0.066 | -0.147 |
| **Alcohol**^§^ | -0.235^*^ | -0.216 | -0.195 | -0.072 | 0.113 | - | -0.026 | 0.039 | -0.163 | -0.060 | 0.090 | -0.134 | -0.075 | -0.173 | -0.161 | -0.100 | 0.072 | -0.040 |
| **n-3 FA suppl.** ^§^ | 0.050 | -0.069 | 0.138 | -0.043 | 0.091 | -0.026 | - | 0.936^**^ | 0.168 | 0.183 | -0.221 | 0.165 | 0.211 | 0.042 | 0.056 | 0.358^**^ | 0.233 | 0.323^**^ |
| **n-3 FA /Zn suppl.** | 0.112 | -0.069 | 0.191 | -0.164 | 0.077 | 0.039 | 0.936^**^ | - | 0.188 | 0.214 | -0.196 | 0.208 | 0.227^*^ | 0.080 | 0.069 | 0.400^**^ | 0.224 | 0.359^**^ |
| **BW** | 0.213 | 0.034 | 0.047 | -0.494^**^ | -0.072 | 0.067 | 0.291^*^ | 0.330^**^ | 0.352^**^ | 0.464^**^ | -0.490^**^ | 0.286^*^ | 0.513^**^ | -0.111 | 0.065 | 0.356^**^ | 0.446^**^ | 0.370^**^ |
| **BMI** | 0.308^**^ | 0.111 | 0.108 | -0.558^**^ | -0.134 | 0.089 | 0.326^**^ | 0.362^**^ | 0.408^**^ | 0.566^**^ | -0.451^**^ | 0.371^**^ | 0.579^**^ | -0.039 | 0.026 | 0.452^**^ | 0.414^**^ | 0.402^**^ |
| **FM%** | 0.281^*^ | 0.108 | 0.051 | -0.614^**^ | -0.112 | 0.085 | 0.203 | 0.266^*^ | 0.328^**^ | 0.480^**^ | -0.365^**^ | 0.339^**^ | 0.486^**^ | -0.031 | 0.134 | 0.326^**^ | 0.448^**^ | 0.344^**^ |
| **FM** | 0.264^*^ | 0.082 | 0.051 | -0.590^**^ | -0.100 | 0.082 | 0.247^*^ | 0.303^**^ | 0.351^**^ | 0.494^**^ | -0.431^**^ | 0.331^**^ | 0.517^**^ | -0.065 | 0.110 | 0.350^**^ | 0.464^**^ | 0.367^**^ |
| **FFM** | -0.019 | -0.141 | -0.073 | -0.187 | 0.068 | 0.050 | 0.228^*^ | 0.208 | 0.183 | 0.180 | -0.388^**^ | 0.094 | 0.261^*^ | -0.187 | 0.013 | 0.222 | 0.375^**^ | 0.288^*^ |
| **VFL** | 0.702^**^ | 0.450^**^ | 0.347^**^ | -0.524^**^ | -0.294^**^ | -0.068 | 0.207 | 0.267^*^ | 0.486^**^ | 0.643^**^ | -0.434^**^ | 0.609^**^ | 0.634^**^ | 0.213 | 0.090 | 0.538^**^ | 0.209 | 0.385^**^ |
| **WC** | 0.268^*^ | 0.125 | 0.081 | -0.500^**^ | -0.189 | 0.046 | 0.308^**^ | 0.331^**^ | 0.364^**^ | 0.503^**^ | -0.484^**^ | 0.366^**^ | 0.541^**^ | -0.093 | 0.044 | 0.358^**^ | 0.439^**^ | 0.334^**^ |
| **HC** | 0.174 | 0.063 | 0.004 | -0.524^**^ | -0.017 | 0.132 | 0.264^*^ | 0.309^**^ | 0.353^**^ | 0.431^**^ | -0.393^**^ | 0.241^*^ | 0.457^**^ | -0.136 | -0.035 | 0.278^*^ | 0.414^**^ | 0.327^**^ |
| **WHR** | 0.256^*^ | 0.140 | 0.135 | -0.239^*^ | -0.306^**^ | -0.079 | 0.218 | 0.203 | 0.207 | 0.354^**^ | -0.367^**^ | 0.344^**^ | 0.389^**^ | 0.005 | 0.120 | 0.287^*^ | 0.242 | 0.169 |
| **WHtR** | 0.325^**^ | 0.174 | 0.121 | -0.523^**^ | -0.226 | 0.059 | 0.319^**^ | 0.339^**^ | 0.387^**^ | 0.553^**^ | -0.434^**^ | 0.411^**^ | 0.564^**^ | -0.036 | 0.014 | 0.415^**^ | 0.396^**^ | 0.341^**^ |

^§^ Point-Biserial *r_pb_* correlation coefficients (otherwise - data represent Pearson’s correlation coefficients - *r*).

* *p* < 0.05; correlation is significant at the 0.05 level (2-tailed).

** *p* < 0.01; correlation is significant at the 0.01 level (2-tailed).

(Underlined coefficients represent those which remained significant after the Bonferroni’s corrections for multiple correlations)

Note: FA = fatty acid, BW = Body weight: BMI = Body Mass Index; FM = Fat mass; FM% = Percentage of fat mass; FFM = Fat-free mass; VFL = visceral fat level; WC = waist circumference; HC = hip circumference; WHR = waist to hip ratio; WHtR = waist to height ratio; GLU = glucose: TAG = Triacylglycerols; HDL-C = High Density Lipoprotein-Cholesterol; LDL-C = Low Density Lipoprotein-Cholesterol; TAG/HDL-C = Triacylglycerols/ HDL-C ratio; AST = aspartate aminotransferase; ALT = alanine aminotransferase; ALT/AST = ALT/AST ratio; BP = Blood Pressure; SBP = Systolic Blood Pressure; DBP = Diastolic Blood Pressure; *r* = Pearson’s correlation coefficients; *p* = statistical significance of correlations. Non-normally distributed data were log 10 transformed before analyses.

**Supplementary Table S2. Inter-correlations of FAs profiles and activities of desaturases in erythrocyte PL**

|  | SFA | | MUFA | | | n-6 PUFA | | | | n-3 PUFA | | | | Estimated desaturases activities | | |
| --- | --- | --- | --- | --- | --- | --- | --- | --- | --- | --- | --- | --- | --- | --- | --- | --- |
|  | 16:0 | 18:0 | 16:1n-7 | 18:1n-9 | 18:1n-7 | 18:2n-6 | 20:3n-6 | 20:4n-6 | 22:4n-6 | 18:3n-3 | 20:5n-3 | 22:5n-3 | 22:6n-3 | D9D | D6D | D5D |
|  | *r* | | | | | | | | | | | | | | | |
| 16:0 | - | 0.178 | 0.324^**^ | 0.415^**^ | 0.129 | -0.087 | -0.360^**^ | -0.689^**^ | -0.585^**^ | -0.410^**^ | 0.014 | -0.223 | -0.347^**^ | 0.043 | -0.322^**^ | -0.041 |
| 18:0 | 0.178 | - | -0.214 | -0.196 | 0.195 | -0.161 | -0.386^**^ | -0.399^**^ | -0.321^**^ | -0.245 | -0.085 | -0.240^*^ | -0.164 | -0.279^*^ | -0.311^**^ | 0.145 |
| 16:1n-7 | 0.324^**^ | -0.214 | - | 0.362^**^ | 0.039 | -0.103 | 0.218 | -0.140 | -0.159 | -0.332^*^ | -0.012 | -0.002 | -0.286^*^ | 0.959^**^ | 0.273^*^ | -0.286^*^ |
| 18:1n-9 | 0.415^**^ | -0.196 | 0.362^**^ | - | -0.004 | -0.114 | -0.185 | -0.365^**^ | -0.435^**^ | -0.207 | -0.044 | -0.142 | -0.259^*^ | 0.258^*^ | -0.131 | -0.027 |
| 18:1n-7 | 0.129 | 0.195 | 0.039 | -0.004 | - | -0.222 | -0.113 | -0.131 | 0.010 | -0.165 | -0.143 | -0.185 | 0.009 | 0.003 | -0.002 | 0.034 |
| 18:2n-6 | -0.087 | -0.161 | -0.103 | -0.114 | -0.222 | - | 0.277^*^ | -0.331^**^ | -0.190 | 0.096 | -0.013 | -0.197 | -0.052 | -0.083 | -0.222 | -0.448^**^ |
| 20:3n-6 | -0.360^**^ | -0.386^**^ | 0.218 | -0.185 | -0.113 | 0.277^*^ | - | 0.202 | 0.368^**^ | 0.061 | -0.045 | 0.127 | -0.036 | 0.338^**^ | 0.875^**^ | -0.839^**^ |
| 20:4n-6 | -0.689^**^ | -0.399^**^ | -0.140 | -0.365^**^ | -0.131 | -0.331^**^ | 0.202 | - | 0.731^**^ | 0.234 | -0.209 | 0.248^*^ | 0.092 | 0.058 | 0.372^**^ | 0.364^**^ |
| 22:4n-6 | -0.585^**^ | -0.321^**^ | -0.159 | -0.435^**^ | 0.010 | -0.190 | 0.368^**^ | 0.731^**^ | - | 0.106 | -0.370^**^ | 0.169 | -0.046 | 0.006 | 0.469^**^ | 0.056 |
| 18:3n-3 | -0.410^**^ | -0.245 | -0.332^*^ | -0.207 | -0.165 | 0.096 | 0.061 | 0.234 | 0.106 | - | 0.314 | 0.420^**^ | 0.401^*^ | -0.216 | 0.013 | 0.070 |
| 20:5n-3 | 0.014 | -0.085 | -0.012 | -0.044 | -0.143 | -0.013 | -0.045 | -0.209 | -0.370^**^ | 0.314 | - | 0.346^**^ | 0.680^**^ | -0.016 | -0.039 | -0.072 |
| 22:5n-3 | -0.223 | -0.240^*^ | -0.002 | -0.142 | -0.185 | -0.197 | 0.127 | 0.248^*^ | 0.169 | 0.420^**^ | 0.346^**^ | - | 0.304^**^ | 0.064 | 0.228^*^ | 0.017 |
| 22:6n-3 | -0.347^**^ | -0.164 | -0.286^*^ | -0.259^*^ | 0.009 | -0.052 | -0.036 | 0.092 | -0.046 | 0.401^*^ | 0.680^**^ | 0.304^**^ | - | -0.199 | -0.010 | 0.085 |
| SFA | 0.809^**^ | 0.720^**^ | 0.097 | 0.163 | 0.205 | -0.156 | -0.485^**^ | -0.725^**^ | -0.610^**^ | -0.434^**^ | -0.027 | -0.297^**^ | -0.326^**^ | -0.140 | -0.414^**^ | 0.058 |
| MUFA | 0.444^**^ | -0.157 | 0.410^**^ | 0.978^**^ | 0.195 | -0.165 | -0.193 | -0.386^**^ | -0.428^**^ | -0.246 | -0.064 | -0.173 | -0.261^*^ | 0.300^**^ | -0.113 | -0.032 |
| PUFA | -0.840^**^ | -0.534^**^ | -0.249^*^ | -0.550^**^ | -0.220 | 0.165 | 0.483^**^ | 0.778^**^ | 0.707^**^ | 0.377^*^ | 0.058 | 0.329^**^ | 0.386^**^ | -0.012 | 0.407^**^ | -0.026 |
| n-6 PUFA | -0.770^**^ | -0.502^**^ | -0.156 | -0.484^**^ | -0.211 | 0.205 | 0.524^**^ | 0.815^**^ | 0.784^**^ | 0.250 | -0.284^*^ | 0.142 | 0.011 | 0.066 | 0.428^**^ | -0.045 |
| n-6 PUFA | -0.377^**^ | -0.242^*^ | -0.239^*^ | -0.262^*^ | -0.064 | -0.095 | 0.033 | 0.160 | 0.033 | 0.438^**^ | 0.750^**^ | 0.538^**^ | 0.956^**^ | -0.140 | 0.081 | 0.058 |
| n-6/n-3 PUFA ratio | 0.103 | 0.063 | 0.178 | 0.088 | -0.010 | 0.161 | 0.146 | 0.122 | 0.234^*^ | -0.368^*^ | -0.812^**^ | -0.471^**^ | -0.918^**^ | 0.157 | 0.067 | -0.071 |
| n-3-index  (20:5n-3  + 22:6n-3) | -0.323^**^ | -0.165 | -0.268^*^ | -0.248^*^ | -0.011 | -0.052 | -0.033 | 0.068 | -0.060 | 0.398^*^ | 0.737^**^ | 0.322^**^ | 0.996^**^ | -0.187 | -0.007 | 0.069 |
| D9D  (16:1n-7/16:0) | 0.043 | -0.279^*^ | 0.959^**^ | 0.258^*^ | 0.003 | -0.083 | 0.338^**^ | 0.058 | 0.006 | -0.216 | -0.016 | 0.064 | -0.199 | - | 0.384^**^ | -0.289^*^ |
| D6D  (20:3n-6/18:2n-6) | -0.322^**^ | -0.311^**^ | 0.273^*^ | -0.131 | -0.002 | -0.222 | 0.875^**^ | 0.372^**^ | 0.469^**^ | 0.013 | -0.039 | 0.228^*^ | -0.010 | 0.384^**^ | - | -0.626^**^ |
| D5D  (20:4n-6/20:3n-6) | -0.041 | 0.145 | -0.286^*^ | -0.027 | 0.034 | -0.448^**^ | -0.839^**^ | 0.364^**^ | 0.056 | 0.070 | -0.072 | 0.017 | 0.085 | -0.289^*^ | -0.626^**^ | - |

* *p* < 0.05; correlation is significant at the 0.05 level (2-tailed).

** *p* < 0.01; correlation is significant at the 0.01 level (2-tailed).

(Underlined coefficients represent those which remained significant after the Bonferroni’s corrections for multiple correlations)

Note: FA = fatty acid; PL = phospholipids; SFA = saturated fatty acids; MUFA = monounsaturated fatty acids; PUFA = polyunsaturated fatty acids; 16:0 = palmitic acid; 18:0 = stearic acid; 16:1n-7 = palmitoleic acid; 18:1n-9 = oleic acid; 18:1n-7 = cis-vaccenic acid; 18:2n-6 = linoleic acid; 20:3n-6 = di-homo-gamma-linolenic acid; 20:4n-6 = arachidonic acid; 22:4n-6 = adrenic acid; 18:3n-3 = alpha-linolenic acid; 20:5n-3 = eicosapentaenoic acid; 22:5n-3 = docosapentaenoic acid; 22:6n-3 = docosahexaenoic acid; D9D = delta-9 desaturase (i.e., D9D-16; stearoyl-CoA desaturase 1, SCD-1); D6D = delta-6 desaturase; D5D = delta-5 desaturase; *r* = Pearson’s correlation coefficients; *p* = statistical significance of correlations. Non-normally distributed data were log 10 transformed before analyses. Individual FAs are expressed as a percentage of total identified FAs.

**Supplementary Table S3. Correlations of selected FAs (18:0, 16:1n-7, 20:3n-6 and 20:5n-3**) and desaturase activities with biochemical and clinical (BP) parameters, after adjustment for FM, FM%, or VFL and all other confounders

|  | **MODEL 4 (corrected for FM, age, and all other confounders)** | | | | | | | **MODEL 5 (corrected for FM%, age, and all other confounders)** | | | | | | | **MODEL 6 (corrected for VFL, age, and all other confounders)** | | | | | | |
| --- | --- | --- | --- | --- | --- | --- | --- | --- | --- | --- | --- | --- | --- | --- | --- | --- | --- | --- | --- | --- | --- |
|  | **18:0** | **16:1n-7** | **20:3n-6** | 20:5n-3 | **D9D** | **D6D** | **D5D** | **18:0** | **16:1n-7** | **20:3n-6** | 20:5n-3 | **D9D** | **D6D** | **D5D** | **18:0** | **16:1n-7** | **20:3n-6** | 20:5n-3 | **D9D** | **D6D** | **D5D** |
|  | *r* | | | | | | | *r* | | | | | | | *r* | | | | | | |
| **GLU** | -0.072 | 0.245^*^ | 0.001 | -0.063 | 0.216 | 0.102 | 0.001 | -0.082 | 0.256^*^ | 0.014 | -0.070 | 0.231 | 0.120 | -0.003 | -0.072 | 0.236 | -0.002 | -0.057 | 0.206 | 0.094 | 0.004 |
| **TAG** | -0.065 | 0.041 | 0.131 | -0.127 | -0.010 | 0.153 | -0.189 | -0.083 | 0.069 | 0.167 | -0.144 | 0.023 | 0.183 | -0.220 | -0.090 | 0.024 | 0.148 | -0.114 | -0.016 | 0.165 | -0.186 |
| **HDL-C** | -0.112 | -0.074 | -0.101 | 0.207 | -0.040 | -0.099 | 0.169 | -0.077 | -0.115 | -0.150 | 0.225 | -0.091 | -0.150 | 0.200 | -0.081 | -0.055 | -0.122 | 0.193 | -0.034 | -0.116 | 0.165 |
| **LDL-C** | 0.056 | -0.128 | -0.169 | 0.174 | -0.088 | -0.169 | 0.198 | 0.049 | -0.116 | -0.147 | 0.165 | -0.075 | -0.153 | 0.179 | 0.047 | -0.156 | -0.168 | 0.192 | -0.110 | -0.176 | 0.207 |
| **TAG/HDL-C** | -0.010 | 0.057 | 0.136 | -0.170 | 0.007 | 0.151 | -0.204 | -0.035 | 0.093 | 0.179 | -0.189 | 0.049 | 0.191 | -0.237 | -0.041 | 0.038 | 0.156 | -0.156 | -0.001 | 0.167 | -0.200 |
| **ALT** | 0.124 | 0.162 | -0.206 | 0.184 | 0.145 | -0.076 | 0.175 | 0.136 | 0.139 | -0.222 | 0.192 | 0.118 | -0.099 | 0.185 | 0.131 | 0.175 | -0.209 | 0.178 | 0.153 | -0.076 | 0.173 |
| **AST** | 0.004 | 0.054 | -0.034 | 0.203 | 0.073 | 0.083 | 0.092 | 0.003 | 0.054 | -0.022 | 0.200 | 0.072 | 0.081 | 0.075 | -0.025 | 0.085 | 0.001 | 0.188 | 0.113 | 0.128 | 0.083 |
| **ALT/AST** | -0.079 | 0.140 | -0.131 | 0.412^**^ | 0.074 | -0.122 | 0.023 | -0.090 | 0.155 | -0.107 | 0.397^**^ | 0.092 | -0.097 | 0.008 | -0.088 | 0.126 | -0.127 | 0.426^**^ | 0.062 | -0.122 | 0.028 |
| **SBP** | 0.011 | -0.162 | 0.302^*^ | -0.041 | -0.145 | 0.283^*^ | -0.243 | -0.007 | -0.132 | 0.335^**^ | -0.061 | -0.112 | 0.308^*^ | -0.277^*^ | -0.020 | -0.175 | 0.320^*^ | -0.030 | -0.144 | 0.300^*^ | -0.238 |
| **DBP** | -0.095 | -0.011 | 0.032 | 0.047 | -0.034 | -0.065 | -0.073 | -0.105 | 0.006 | 0.053 | 0.036 | -0.015 | -0.043 | -0.090 | -0.099 | -0.048 | 0.026 | 0.069 | -0.069 | -0.085 | -0.066 |

MODEL 4 - corrected for FM, age, smoking, physical activity, level of education, moderate alcohol consumption, and n-3/Zn supplementation.

MODEL 5 - corrected for FM%, age, smoking, physical activity, level of education, moderate alcohol consumption, and n-3/Zn supplementation.

MODEL 6 - corrected for VFL, age, smoking, physical activity, level of education, moderate alcohol consumption, and n-3/Zn supplementation.

* *p* < 0.05; correlation is significant at the 0.05 level (2-tailed).

** *p* < 0.01; correlation is significant at the 0.01 level (2-tailed).

(Underlined coefficients represent those which remained significant after the Bonferroni’s corrections for multiple correlations)

Note: FA = fatty acid; 18:0 = stearic acid; 16:1n-7 = palmitoleic acid; 20:3n-6 = di-homo-gamma-linolenic; 20:5n-3 = eicosapentaenoic acid; D9D = delta-9 desaturase (i.e., D9D-16; stearoyl-CoA desaturase 1, SCD-1); D6D = delta-6 desaturase; D5D = delta-5 desaturase; FM = Fat mass; FM% = Percentage of fat mass; VFL = visceral fat level; GLU = glucose: TAG = triacylglycerols; HDL-C = High Density Lipoprotein-Cholesterol; LDL-C = Low Density Lipoprotein-Cholesterol; TAG/HDL-C = Triacylglycerols/ HDL-C ratio; AST = aspartate aminotransferase; ALT = alanine aminotransferase; ALT/AST = ALT/AST ratio; BP = Blood Pressure; SBP = Systolic Blood Pressure; DBP = Diastolic Blood Pressure; *r* = Pearson’s correlation coefficients; *p* = statistical significance of correlations. Non-normally distributed data were log 10 transformed before analyses. Individual FAs were expressed as a percentage of total identified FAs.

**Supplementary Table S4. Correlations of selected FAs (18:0, 16:1n-7, 20:3n-6 and 20:5n-3**) and desaturase activities with biochemical and clinical (BP) parameters, after adjustment for WC, HC, or WHtR and all other confounders

|  | **MODEL 4 (corrected for WC, age, and all other confounders)** | | | | | | | **MODEL 5 (corrected for HC, age, and all other confounders)** | | | | | | | **MODEL 6 (corrected for WHtR, age, and all other confounders)** | | | | | | |
| --- | --- | --- | --- | --- | --- | --- | --- | --- | --- | --- | --- | --- | --- | --- | --- | --- | --- | --- | --- | --- | --- |
|  | **18:0** | **16:1n-7** | **20:3n-6** | 20:5n-3 | **D9D** | **D6D** | **D5D** | **18:0** | **16:1n-7** | **20:3n-6** | 20:5n-3 | **D9D** | **D6D** | **D5D** | **18:0** | **16:1n-7** | **20:3n-6** | 20:5n-3 | **D9D** | **D6D** | **D5D** |
|  | *r* | | | | | | | *r* | | | | | | | *r* | | | | | | |
| **GLU** | -0.078 | 0.240^*^ | -0.009 | -0.055 | 0.211 | 0.090 | 0.011 | -0.059 | 0.214 | -0.018 | -0.042 | 0.181 | 0.074 | 0.008 | -0.084 | 0.239^*^ | -0.006 | -0.053 | 0.211 | 0.092 | 0.015 |
| **TAG** | -0.119 | 0.082 | 0.122 | -0.106 | 0.047 | 0.145 | -0.158 | -0.082 | 0.038 | 0.151 | -0.110 | -0.005 | 0.186 | -0.190 | -0.148 | 0.059 | 0.125 | -0.089 | 0.039 | 0.144 | -0.135 |
| **HDL-C** | -0.048 | -0.116 | -0.082 | 0.180 | -0.098 | -0.076 | 0.129 | -0.087 | -0.070 | -0.126 | 0.187 | -0.047 | -0.141 | 0.170 | -0.014 | -0.111 | -0.111 | 0.177 | -0.111 | -0.117 | 0.117 |
| **LDL-C** | 0.026 | -0.109 | -0.186 | 0.196 | -0.061 | -0.192 | 0.226 | 0.037 | -0.115 | -0.142 | 0.174 | -0.068 | -0.127 | 0.191 | 0.009 | -0.124 | -0.183 | 0.209 | -0.065 | -0.187 | 0.244^*^ |
| **TAG/HDL-C** | -0.075 | 0.103 | 0.122 | -0.144 | 0.070 | 0.137 | -0.166 | -0.032 | 0.053 | 0.159 | -0.149 | 0.012 | 0.190 | -0.204 | -0.107 | 0.085 | 0.134 | -0.131 | 0.069 | 0.151 | -0.144 |
| **ALT** | 0.140 | 0.157 | -0.189 | 0.168 | 0.135 | -0.047 | 0.157 | 0.122 | 0.187 | -0.202 | 0.170 | 0.169 | -0.070 | 0.173 | 0.151 | 0.155 | -0.202 | 0.171 | 0.125 | -0.069 | 0.156 |
| **AST** | -0.031 | 0.094 | -0.008 | 0.192 | 0.122 | 0.122 | 0.092 | -0.035 | 0.115 | 0.018 | 0.174 | 0.146 | 0.157 | 0.075 | -0.037 | 0.099 | 0.003 | 0.188 | 0.129 | 0.137 | 0.090 |
| **ALT/AST** | -0.105 | 0.161 | -0.128 | 0.418^**^ | 0.103 | -0.115 | 0.035 | -0.093 | 0.151 | -0.111 | 0.413^**^ | 0.089 | -0.089 | 0.019 | -0.117 | 0.147 | -0.136 | 0.439^**^ | 0.094 | -0.127 | 0.052 |
| **SBP** | -0.051 | -0.105 | 0.297^*^ | -0.024 | -0.070 | 0.284^*^ | -0.210 | -0.020 | -0.138 | 0.325^*^ | -0.036 | -0.109 | 0.327^*^ | -0.241 | -0.076 | -0.111 | 0.311^*^ | -0.020 | -0.061 | 0.303^*^ | -0.198 |
| **DBP** | -0.125 | 0.025 | 0.047 | 0.045 | 0.012 | -0.040 | -0.067 | -0.106 | -0.008 | 0.047 | 0.053 | -0.026 | -0.038 | -0.076 | -0.134 | 0.020 | 0.050 | 0.049 | 0.012 | -0.035 | -0.061 |

MODEL 4 - corrected for WC, age, smoking, physical activity, level of education, moderate alcohol consumption, and n-3/Zn supplementation.

MODEL 5 - corrected for HC, age, smoking, physical activity, level of education, moderate alcohol consumption, and n-3/Zn supplementation.

MODEL 6 - corrected for WHtR, age, smoking, physical activity, level of education, moderate alcohol consumption, and n-3/Zn supplementation.

* *p* < 0.05; correlation is significant at the 0.05 level (2-tailed).

** *p* < 0.01; correlation is significant at the 0.01 level (2-tailed).

(Underlined coefficients represent those which remained significant after the Bonferroni’s corrections for multiple correlations)

Note: FA = fatty acid; 18:0 = stearic acid; 16:1n-7 = palmitoleic acid; 20:3n-6 = di-homo-gamma-linolenic; 20:5n-3 = eicosapentaenoic acid; D9D = delta-9 desaturase (i.e., D9D-16; stearoyl-CoA desaturase 1, SCD-1); D6D = delta-6 desaturase; D5D = delta-5 desaturase; WC = waist circumference; HC = hip circumference; WHtR = waist to height ratio; GLU = glucose: TAG = triacylglycerols; HDL-C = High Density Lipoprotein-Cholesterol; LDL-C = Low Density Lipoprotein-Cholesterol; TAG/HDL-C = Triacylglycerols/ HDL-C ratio; AST = aspartate aminotransferase; ALT = alanine aminotransferase; ALT/AST = ALT/AST ratio; BP = Blood Pressure; SBP = Systolic Blood Pressure; DBP = Diastolic Blood Pressure; *r* = Pearson’s correlation coefficients; *p* = statistical significance of correlations. Non-normally distributed data were log 10 transformed before analyses. Individual FAs were expressed as a percentage of total identified FAs.

**Supplementary Table S5. Linear regression models (method: enter) for predicting proportions of 18:0, 16:1n-7, 20:3n-6, 20:5n-3 and desaturases activities using only BMI as a predictor**

| Dependent variable | Model | Adjusted *R^2^* | ANOVA *p* | Coefficients | *β* | *B* | 95% *CI* for *B* |
| --- | --- | --- | --- | --- | --- | --- | --- |
|  |  |  |  |  |  |  | Lower Bound  Upper Bound |
| 18:0 | 2 | 0.074 | 0.010 | (Constant) |  | 1.459 | [1.351, 1.567]** |
|  |  |  |  | BMI | -0.295 | -0.105 | [-0.184, -0.026]* |
| 16:1n-7 | 2 | 0.213 | <0.001 | (Constant) |  | -1.539 | [-1.937, -1.141] ** |
|  |  |  |  | BMI | 0.473 | 0.676 | [0.384, 0.967]** |
| 20:3n-6 | 2 | 0.178 | <0.001 | (Constant) |  | -0.387 | [-0.683, -0.090]* |
|  |  |  |  | BMI | 0.435 | 0.453 | [0.236, 0.671]** |
| 20:5n-3 | 2 | -0.009 | 0.546 | (Constant) |  | -0.837 | [-1.524, -0.150]* |
|  |  |  |  | BMI | 0.071 | 0.153 | [-0.350, 0.656] |
| D9D | 2 | 0.247 | <0.001 | (Constant) |  | 0.119 | [-0.250, 0.488] |
|  |  |  |  | BMI | 0.507 | 0.686 | [0.415, 0.956]** |
| D6D | 2 | 0.250 | <0.001 | (Constant) |  | 0.384 | [0.105, 0.664]** |
|  |  |  |  | BMI | 0.510 | 0.523 | [0.319, 0.728]** |
| D5D | 2 | 0.103 | 0.003 | (Constant) |  | 1.508 | [1.182, 1.834]** |
|  |  |  |  | BMI | -0.339 | -0.372 | [-0.611, -0.133]** |

MODEL 2 – predictors: only BMI (excluded others)

* *p* < 0.05; regression coefficients are significant at the 0.05 level (2-tailed.

** *p* < 0.01; regression coefficients are significant at the 0.01 level (2-tailed).

Note: 18:0 = stearic acid; 16:1n-7 = palmitoleic acid; 20:3n-6 = di-homo-gamma-linolenic acid; 20:5n-3 = eicosapentaenoic acid; D9D = delta-9 desaturase (i.e., D9D-16; stearoyl-CoA desaturase 1, SCD-1); D6D = delta-6 desaturase; D5D = delta-5 desaturase; BMI = Body Mass Index; ANOVA = analysis of variance; *R^2^* = coefficient of determination; *β* (Beta) = standardized linear regression coefficients; *B* = unstandardized linear regression coefficients; *CI* = confidence intervals; *p* = statistical significance. Individual FAs are expressed as a percentage of total identified FAs.

**Supplementary Table S6. Linear (stepwise) regression models for predicting proportions of 20:4n-6 by 18:2n-6**, D6D and D5D

| Dependent variable | Model | Adjusted *R^2^* | ANOVA *p* | Coefficients | *β* | *B* | 95% *CI* for *B* |
| --- | --- | --- | --- | --- | --- | --- | --- |
|  |  |  |  |  |  |  | Lower Bound  Upper Bound |
| 20:4n-6 | 1 | 0.126 | 0.001 | (Constant) |  | 0.990 | [0.850, 1.131]** |
|  |  |  |  | D6D | 0.372 | 0.221 | [0.093, 0.348] ** |
|  | 2 | 0.716 | <0.001 | (Constant) |  | 0.044 | [-0.127, 0.216] |
|  |  |  |  | D6D | 0.986 | 0.585 | [0.491, 0.678] ** |
|  |  |  |  | D5D | 0.981 | 0.545 | [0.458, 0.633] ** |
|  | 3 | 1.000 | <0.001 | (Constant) |  | -2.001 | [-2.002, -2.000] ** |
|  |  |  |  | D6D | 1.686 | 1.000 | [1.000, 1.000] ** |
|  |  |  |  | D5D | 1.798 | 1.000 | [1.000, 1.000] ** |
|  |  |  |  | 18:2n-6 | 0.848 | 1.000 | [1.000, 1.001] ** |

* *p* < 0.05; regression coefficients are significant at the 0.05 level (2-tailed.

** *p* < 0.01; regression coefficients are significant at the 0.01 level (2-tailed).

Note: 20:4n-6 = arachidonic acid; D6D = delta-6 desaturase; D5D = delta-5 desaturase; 18:2n-6 = linoleic acid; ANOVA = analysis of variance; *R^2^* = coefficient of determination; *β* (Beta) = standardized linear regression coefficients; *B* = unstandardized linear regression coefficients; *CI* = confidence intervals; *p* = statistical significance. Individual FAs are expressed as a percentage of total identified FAs.

**Supplementary Table S7.** **Linear (stepwise) regression models for prediction of biochemical data by FAs and desaturases activities in erythrocytes PL**

| Dependent variable | Model | Adjusted *R^2^* | ANOVA *p* | Coefficients | *β* | *B* | 95% *CI* for *B* |
| --- | --- | --- | --- | --- | --- | --- | --- |
|  |  |  |  |  |  |  | Lower Bound  Upper Bound |
| GLU | 1 | 0.214 | 0.003 | (Constant) |  | 0.648 | [0.624, 0.671]** |
|  |  |  |  | VFL | 0.486 | 0.063 | [0.023, 0.102]** |
| TAG | 1 | 0.396 | <0.001 | (Constant) |  | -0.231 | [-0.328, -0.134]** |
|  |  |  |  | VFL | 0.643 | 0.384 | [0.225, 0.544]** |
| HDL-C | 1 | 0.212 | 0.003 | (Constant) |  | 1.627 | [0.729, 2.526]** |
|  |  |  |  | Waist circumference | -0.484 | -0.747 | [-1.217, -0.277]** |
| LDL-C | 1 | 0.400 | <0.001 | (Constant) |  | -0.592 | [-1.014, -0.171]** |
|  |  |  |  | Age | 0.646 | 0.663 | [0.390, 0.935]** |
| TAG/HDL-C | 1 | 0.384 | <0.001 | (Constant) |  | -0.481 | [-0.607, -0.354]** |
|  |  |  |  | VFL | 0.634 | 0.489 | [0.281, 0.697]** |
| ALT | 1 | 0.353 | <0.001 | (Constant) |  | -0.299 | [-0.988, 0.390] |
|  |  |  |  | Age | 0.605 | 0.621 | [0.327, 0.915]** |
|  |  |  |  | 18:3n-3 | -0.372 | -0.461 | [-0.816, -0.106]* |
| ALT/AST | 1 | 0.458 | <0.001 | (Constant) |  | 0.016 | [-0.122, 0.153] |
|  |  |  |  | VFL | 0.471 | 0.165 | [0.070, 0.260]** |
|  |  |  |  | 20:5n-3 | 0.385 | 0.266 | [0.079, 0.453]** |
|  |  |  |  | 16:1n-7 | 0.259 | 0.271 | [0.048, 0.494]* |

MODEL 1– predictors: age, postmenopausal status, smoking, physical activity, level of education, moderate alcohol consumption, n-3/Zn supplementation, total energy intakes and specific macronutrients intakes: carbohydrates, proteins, total fats, SFA, MUFA, n-6 PUFA, n-3 PUFA, TFA and cholesterol per day, FAs composition and desaturase activities in erythrocytes PL, and all anthropometric indices

* *p* < 0.05; regression coefficients are significant at the 0.05 level (2-tailed.

** *p* < 0.01; regression coefficients are significant at the 0.01 level (2-tailed).

Note: FA = fatty acids; PL = phospholipids; GLU = glucose: TAG = Triacylglycerols; HDL-C = High Density Lipoprotein-Cholesterol; LDL-C = Low Density Lipoprotein-Cholesterol; TAG/HDL-C = Triacylglycerols/ HDL-C ratio; ALT = alanine aminotransferase; ALT/AST = ALT/AST ratio; BMI = Body Mass Index; VFL = visceral fat level; 18:3n-3 = alpha-linolenic acid; 20:5n-3 = eicosapentaenoic acid; 16:1n-7 = palmitoleic acid; ANOVA = analysis of variance; *R^2^* = coefficient of determination; *β* (Beta) = standardized linear regression coefficients; *B* = unstandardized linear regression coefficients; *CI* = confidence intervals; *p* = statistical significance. Individual FAs were expressed as a percentage of total identified FAs.

**Supplementary Table S8. Mean values of selected FAs and desaturases activities in different BMI-categories and statistical difference between them by ANOVA**

|  | Underweight  N = 7 | | Normal-weight  N = 50 | | Overweight  N = 9 | | Obese  N = 10 | | ANOVA |
| --- | --- | --- | --- | --- | --- | --- | --- | --- | --- |
|  | *Mean* | *SD* | *Mean* | *SD* | *Mean* | *SD* | *Mean* | *SD* | *p* |
| 18:0 | 21.8 | 2.7 | 20.8 | 1.5 | 20.4 | 1.4 | 20.0 | 0.7 | NS |
| 16:1n-7 | 0.20 | 0.11 | 0.24 | 0.07 | 0.25 | 0.03 | 0.33 | 0.11 | 0.002** |
| 18:2n-6 | 14.2 | 1.1 | 13.6 | 1.4 | 13.9 | 2.0 | 13.4 | 1.5 | NS |
| 20:3n-6 | 1.5 | 0.3 | 1.7 | 0.4 | 1.8 | 0.3 | 2.2 | 0.5 | 0.001** |
| 20:4n-6 | 15.4 | 2.3 | 17.4 | 2.2 | 17.7 | 1.8 | 17.0 | 1.1 | NS |
| 20:5n-3 | 0.37 | 0.29 | 0.24 | 0.10 | 0.21 | 0.09 | 0.34 | 0.19 | NS |
| D9D activity (multiplied by 1000) | 9.0 | 4.3 | 11.4 | 2.7 | 11.8 | 1.3 | 15.4 | 4.7 | <0.001** |
| D6D activity (multiplied by 100) | 10.8 | 1.3 | 12.3 | 2.6 | 13.2 | 1.8 | 16.4 | 3.8 | <0.001** |
| D5D activity | 10.2 | 2.7 | 10.8 | 2.1 | 10.0 | 2.1 | 8.1 | 1.9 | 0.002** |

* *p* < 0.05; ANOVA *p* significant at the 0.05 level (2-tailed).

** *p* < 0.01; ANOVA *p* significant at the 0.01 level (2-tailed).

Note: FA = fatty acids; BMI = Body Mass Index; 18:0 = stearic acid; 16:1n-7 = palmitoleic acid; 18:2n-6 = linoleic acid; 20:3n-6 = di-homo-gamma-linolenic acid; 20:4n-6 = arachidonic acid; 20:5n-3 = eicosapentaenoic acid; D9D = delta-9 desaturase; D6D = delta-6 desaturase; D5D = delta-5 desaturase; *SD* = standard deviation; ANOVA = analysis of variance; *p* = statistical significance. Non-normally distributed data were log 10 transformed before analyses. Individual FAs were expressed as a percentage of total identified FAs.

**Supplementary Table S9. Mean values of selected FAs and desaturases activities in different BMI-quartiles and statistical difference between them by ANOVA (with *post-hoc* tests)**

|  | Q1  N = 19  (BMI 16.2 - 20.0) | | Q2  N = 19  (BMI 20.1 - 21.6) | | Q2  N = 19  (BMI 21.8 - 24.7) | | Q4  N = 19  (BMI 26.3 - 43.2) | | ANOVA | *Post-hoc* tests | | | | | |
| --- | --- | --- | --- | --- | --- | --- | --- | --- | --- | --- | --- | --- | --- | --- | --- |
|  |  |  |  |  |  |  |  |  |  | Q1-Q2 | Q2-Q3 | Q1-Q3 | Q1-Q4 | Q2-Q4 | Q3-Q4 |
|  | *Mean* | *SD* | *Mean* | *SD* | *Mean* | *SD* | *Mean* | *SD* | *p* | *p* | *p* | *p* | *p* | *p* | *p* |
| BMI^¥^ | 18.6 | 1.2 | 20.7 | 0.5 | 23.4 | 0.9 | 31.6 | 5.1 | <0.001** | <0.001** | <0.001** | <0.001** | <0.001** | <0.001** | <0.001** |
| 18:0^¥^ | 21.5 | 2.2 | 20.9 | 1.2 | 20.5 | 1.5 | 20.2 | 1.1 | NS | NS | NS | NS | NS | NS | NS |
| 16:1n-7 | 0.21 | 0.08 | 0.24 | 0.08 | 0.26 | 0.05 | 0.29 | 0.09 | 0.002** | NS | NS | 0.026* | 0.002** | NS | NS |
| 18:2n-6 | 14.0 | 1.4 | 14.0 | 1.0 | 13.1 | 1.5 | 13.7 | 1.7 | NS | NS | NS | NS | 0.886 | NS | NS |
| 20:3n-6 | 1.7 | 0.4 | 1.7 | 0.3 | 1.6 | 0.4 | 2.0 | 0.4 | 0.007** | NS | NS | NS | 0.022* | 0.039* | 0.012* |
| 20:4n-6 | 16.5 | 2.4 | 17.2 | 2.2 | 17.7 | 2.2 | 17.3 | 1.5 | NS | NS | NS | NS | 0.555 | NS | NS |
| 20:5n-3 | 0.27 | 0.20 | 0.24 | 0.11 | 0.27 | 0.10 | 0.28 | 0.16 | NS | NS | NS | NS | 0.983 | NS | NS |
| D9D activity (multiplied by 1000) ^¥^ | 9.9 | 3.1 | 11.1 | 3.3 | 12.3 | 2.1 | 13.7 | 3.9 | 0.001** | NS | NS | 0.032* | 0.007** | NS | NS |
| D6D activity (multiplied by 100) | 11.9 | 2.7 | 12.0 | 2.1 | 12.5 | 2.7 | 14.9 | 3.4 | 0.003** | NS | NS | NS | 0.006** | 0.010* | 0.046* |
| D5D activity | 10.4 | 2.3 | 10.6 | 2.0 | 11.2 | 2.2 | 9.0 | 2.2 | 0.015* | NS | NS | NS | NS | NS | 0.011* |

^¥^ the Games Howell (G-H) *post hoc* test, otherwise the Tukey's honestly significant difference (HSD) *post hoc* test

* *p* < 0.05; *p* significant at the 0.05 level (2-tailed).

** *p* < 0.01; *p* significant at the 0.01 level (2-tailed).

Note: FA = fatty acids; BMI = Body Mass Index; 18:0 = stearic acid; 16:1n-7 = palmitoleic acid; 18:2n-6 = linoleic acid; 20:3n-6 = di-homo-gamma-linolenic acid; 20:4n-6 = arachidonic acid; 20:5n-3 = eicosapentaenoic acid; D9D = delta-9 desaturase; D6D = delta-6 desaturase; D5D = delta-5 desaturase; *SD* = standard deviation; ANOVA = analysis of variance; *p* = statistical significance; NS = non-significant. Non-normally distributed data were log 10 transformed before analyses. Individual FAs were expressed as a percentage of total identified FAs.

## Supplementary Figures

**Supplementary figure S1. Typical chromatogram of isolated FAME in erythrocytes PL**


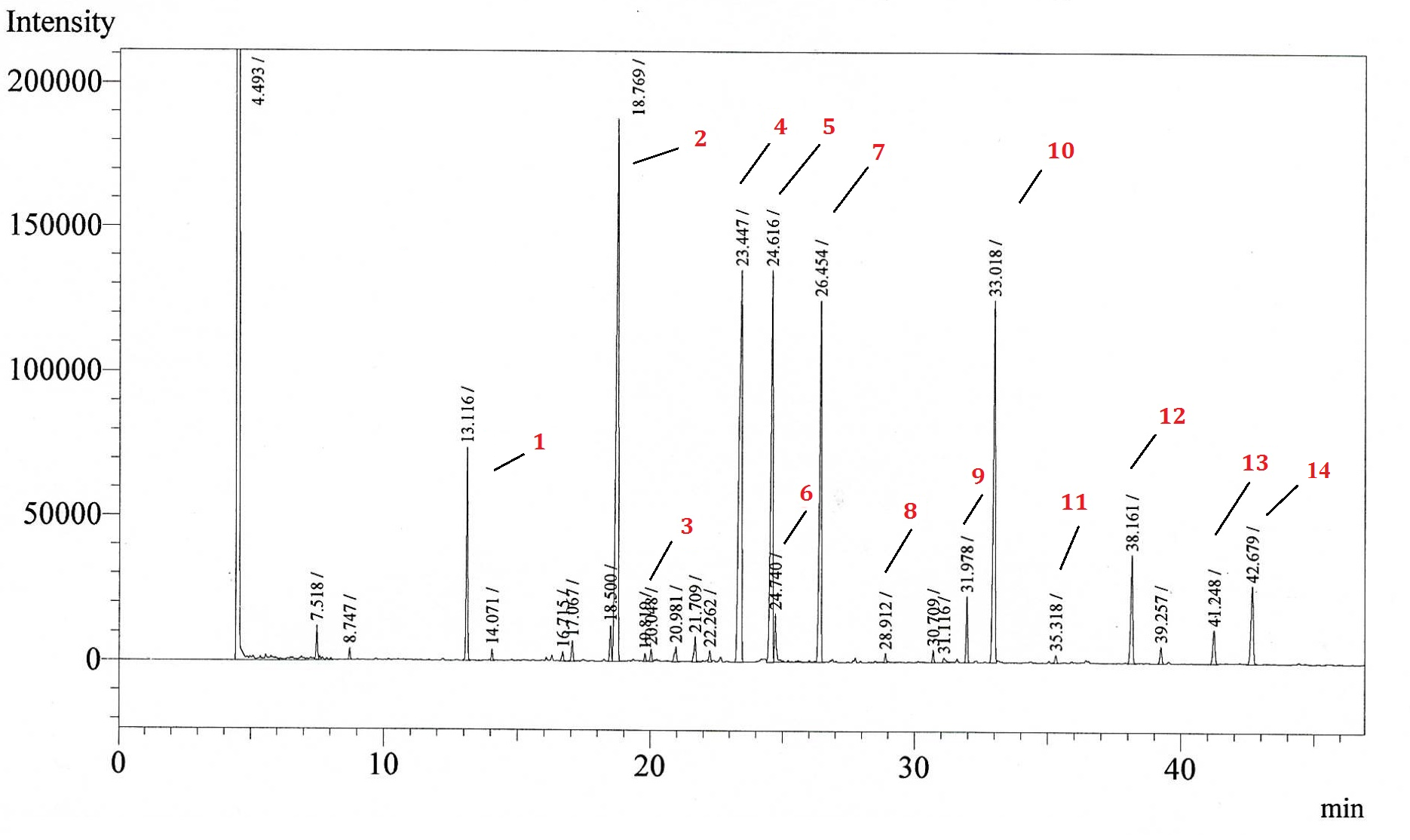


Figure legend:

BHT (2,6-di-tert-butyl-4-methylphenol)

Palmitic Acid Methyl Ester (16:0);

Palmitoleic Acid Methyl Ester (16:1n-7)

Stearic Acid Methyl Ester (18:0)

Oleic Acid Methyl Ester (18:1n-9c)

cis-11-Octadecenoic Acid (cis-Vaccenic Acid) Methyl Ester (18:1n-7c)

Linoleic Acid Methyl Ester (18:2n-6c)

α-Linolenic Acid Methyl Ester (18:3n-3)

cis-8,11,14-Eicosatrienoic Acid (Di-Homo-γ-Linolenic Acid) Methyl Ester (20:3n-6)

Arachidonic Acid Methyl Ester (20:4n-6)

cis-5,8,11,14,17-Eicosapentaenoic Acid Methyl Ester (20:5n-3)

cis-7,10,13,16-Docosatetraenoic Acid Methyl Ester (22:4n-6)

cis-7,10,13,16,19-Docosapentaenoic Acid Methyl Ester (22:5n-3)

cis-4,7,10,13,16,19-Docosahexaenoic Acid Methyl Ester (22:6n-3)

**Supplementary figure S2. Mean values (± *SD*) of desaturases activities (panel A) and selected FAs (18:0, 16:1n-7, 18:2n-6, 20:3n-6, 20:4n-6 and 20:5n-3) (panels B and C) across different BMI- quartiles (Q1-Q4)**


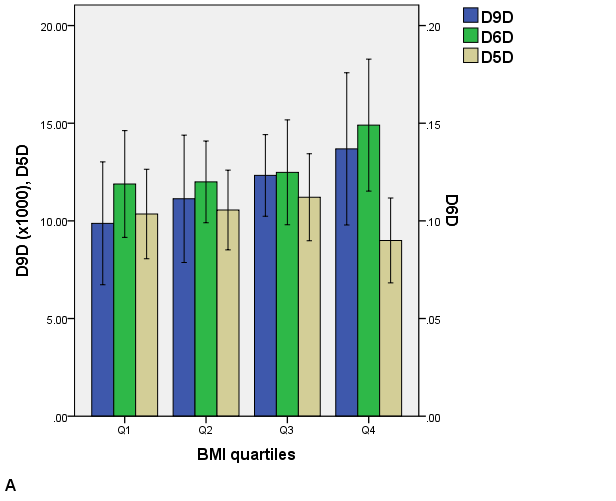

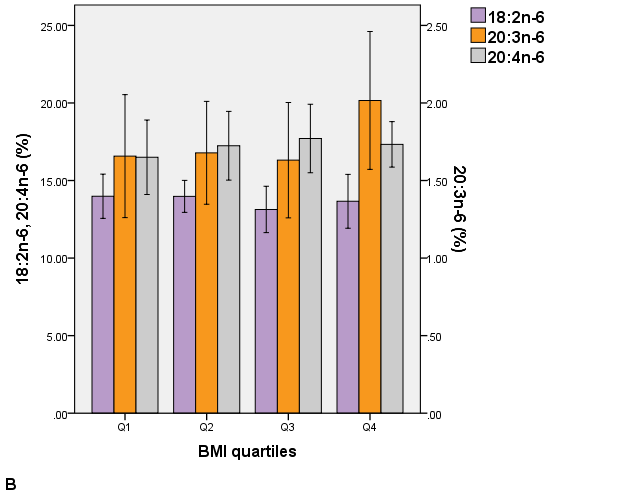

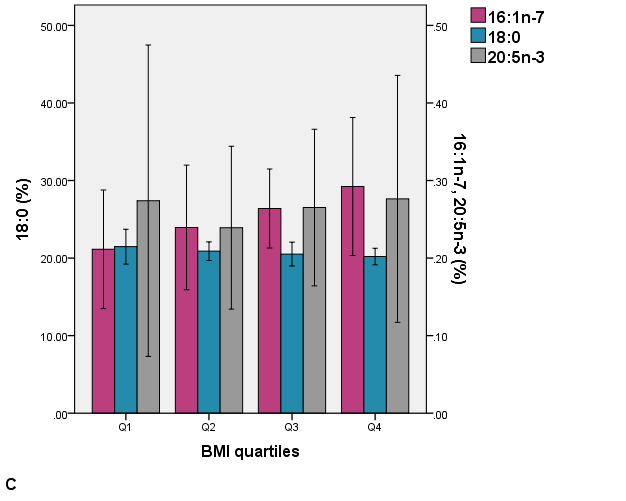


Note: FA = fatty acids; BMI = Body Mass Index; *SD* = standard deviation; Panel (A): D9D = delta-9 desaturase; D6D = delta-6 desaturase; D5D = delta-5 desaturase; Panel (B): 18:2n-6 = linoleic acid; 20:3n-6 = di-homo-gamma-linolenic acid; 20:4n-6 = arachidonic acid; Panel (C): 16:1n-7 = palmitoleic acid; 18:0 = stearic acid; 20:5n-3 = eicosapentaenoic acid. Individual FAs were expressed as a percentage of total identified FAs. Values represent *Mean* ± *SD.* Individual FAs were expressed as a percentage of total identified FAs.

**Supplementary figure S3. Means plots of desaturases activities across different BMI-quartiles (Q1-Q4)**


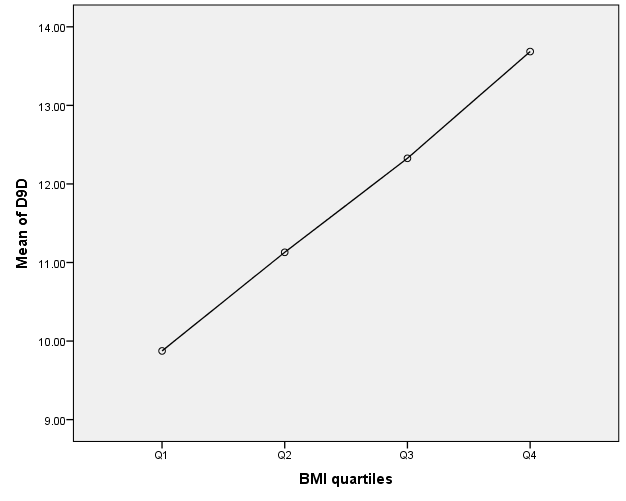

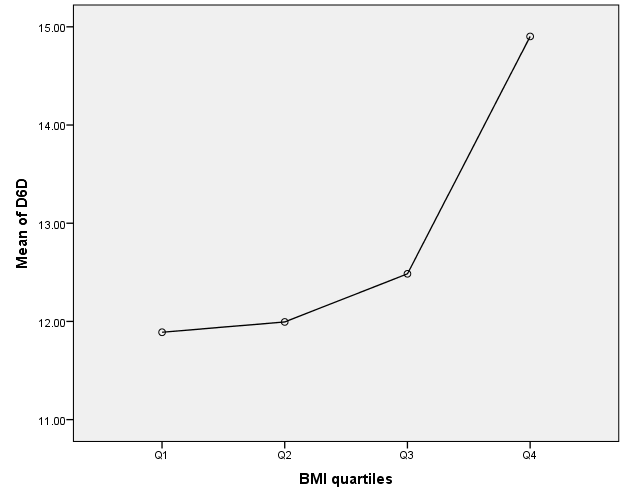

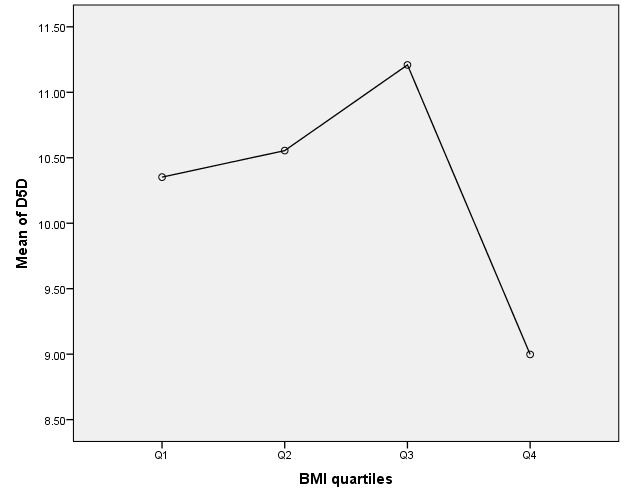


Note: BMI = Body Mass Index; D9D = delta-9 desaturase; D6D = delta-6 desaturase; D5D = delta-5 desaturase.

**Supplementary figure S4. Scatter plot and regression lines of D5D activities across different BMI-quartiles (Q1-Q4): locally weighted scatter plot smoothing regression line (Panel A) and linear regression lines across different BMI-quartiles (Panel B)**

A
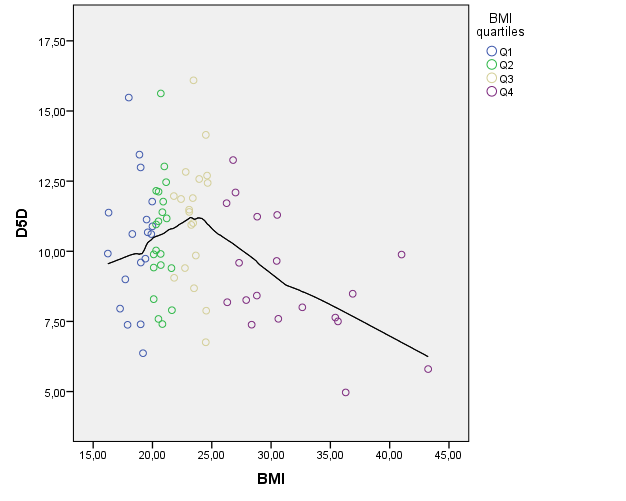
B
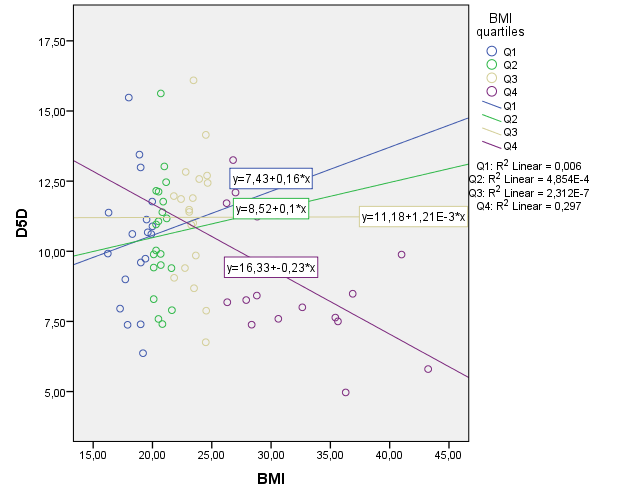


Note: BMI = Body Mass Index; D5D = delta-5 desaturase. Panel (A): Locally weighted scatter plot smoothing regression line with the Epanechnikov kernel permits; Panel (B): Linear regression lines across different BMI-quartiles

**Supplementary Table S10. Linear regression models for predicting D5D activity across different BMI-quartiles, using all variables as predictors (MODEL 1**) or only BMI as a predictor (MODEL 2)

| BMI-quartile | Model | Adjusted *R^2^* | ANOVA *p* | Predictor | *β* | *B* | 95% *CI* for *B* |
| --- | --- | --- | --- | --- | --- | --- | --- |
|  |  |  |  |  |  |  | Lower Bound  Upper Bound |
| Q1 | 1 | - |  | BMI | - | - | - |
|  | 2 | 0.007 | 0.744 | BMI | 0.083 | 0.291 | [-1.560, 2.141] |
| Q2 | 1 | - | - | BMI | - | - | - |
|  | 2 | -0.055 | 0.967 | BMI | 0.010 | 0.086 | [-4.263, 4.435] |
| Q3 | 1 | 0.219 | 0.033 | BMI | - | - | - |
|  | 2 | -0.056 | 0.839 | BMI | -0.050 | -0.276 | [-3.092. 2.540] |
| Q4 | 1 | 0.487 | 0.002* | BMI | -0.456 | -0.742 | [-1.,341, -0.142]* |
|  | 2 | 0.286 | 0.011* | BMI | -0.571 | -0.928 | [-1.611, -0.245]* |

MODEL 1– predictors: BMI, age, postmenopausal status, smoking, physical activity, level of education, moderate alcohol consumption, n-3/Zn supplementation, total energy intakes and specific macronutrients intakes: carbohydrates, proteins, total fats, SFA, MUFA, n-6 PUFA, n-3 PUFA, TFA and cholesterol per day; method: stepwise

MODEL 2 – predictors: only BMI (excluded others); method: enter

* *p* < 0.05; regression coefficients are significant at the 0.05 level (2-tailed.

Note: D5D = delta-5 desaturase; BMI = Body Mass Index; ANOVA = analysis of variance; *R^2^* = coefficient of determination; *β* (Beta) = standardized linear regression coefficients; *B* = unstandardized linear regression coefficients; *CI* = confidence intervals; *p* = statistical significance. Individual FAs are expressed as a percentage of total identified FAs.
